# Pomegranate Supplementation Accelerates Recovery of Muscle Damage and Soreness and Inflammatory Markers after a Weightlifting Training Session

**DOI:** 10.1371/journal.pone.0160305

**Published:** 2016-10-20

**Authors:** Achraf Ammar, Mouna Turki, Hamdi Chtourou, Omar Hammouda, Khaled Trabelsi, Choumous Kallel, Osama Abdelkarim, Anita Hoekelmann, Mohamed Bouaziz, Fatma Ayadi, Tarak Driss, Nizar Souissi

**Affiliations:** 1 Research Unit: Education, Motricité, Sport et Santé, UR15JS01, High Institute of Sport and Physical Education of Sfax, University of Sfax, Sfax, Tunisia; 2 Institute of Sport Science, Otto-von-Guericke-University Magdeburg, Magdeburg, Germany; 3 Laboratory of Biochemistry, CHU Habib Bourguiba, Sfax University, Sfax, Tunisia; 4 High Institute of Sport and Physical Education, Sfax University, Sfax, Tunisia; 5 Research Center on Sport and Movement (EA 2931), University of Paris Ouest La Defense, Nanterre, France; 6 Laboratory of Pharmacology, Faculty of Medicine, Sfax University, Sfax, Tunisia; 7 Laboratory of Hematology, CHU Habib Bourguiba, Sfax, Tunisia; 8 Electrochemistry and Environment Laboratory, ENIS, Sfax University, Sfax, Tunisia; 9 High Institute of Biotechnology, Sfax University, Sfax, Tunisia; 10 National Observatory of Sport, Tunis, Tunisia; University of Alabama at Birmingham, UNITED STATES

## Abstract

**Purpose:**

The aim of this study was to investigate the effect of natural Pomegranate juice supplementation on performance and acute and delayed responses of muscle soreness and biomarkers of muscle damage after a weightlifting training session.

**Methods:**

Nine elite weightlifters (21±0.5 years) performed two Olympic-Weightlifting-sessions after either placebo (PLA) or natural pomegranate juice (POMj) supplementations. Heart rate, blood pressure and blood samples (hematological parameters, muscle damage and C-reactive protein (CRP)) were collected at rest, 3min and 48h after each session. Weightlifting performance, RPE, and DOMS were also assessed after each training session.

**Results:**

T-test showed higher performance (+8.30%) and lower RPE values (-4.37%) using POMj supplementation (p<0.05) in comparison with PLA. For the DOMS values, a significant improvement (13.4%) was shown only for the knee extensors (p<0.01) using the POMj. Compared to PLA condition, POMj attenuated the acute (i.e., 3min) increase of systolic blood pressure (SBP), HR, CK and LDH (p<0.05; -4.46%, -1.81%, -8.75%, -1.64%, respectively) and blunted the significant increase of ASAT, PAL and CRP (p>0.05). Additionally, during the 48h following the training session, POMj improved the recovery kinetic of SBP (p<0.01, 7.97%), CK (p<0.001, 11.34%), LDH (p<0.05, 7.30%) and ASAT (p<0.05, 6.77%). Indeed, the present study showed that 48h of recovery associated to natural POMj supplementation was sufficient to reach the resting values of the selected muscle damage markers after intensive training session.

**Conclusion:**

Natural POMj seems to ameliorate the capacity to adhere to an intensive training program. Therefore, elite weightlifters are advised to use natural POMj during intensive training program and competition to accelerate muscle recovery.

**Trial Registration:**

ClinicalTrials.gov NCT02697903

## Introduction

Exhaustive or unaccustomed intense exercise can decrease maximal strength [1], and increase muscle damage, oxidative stress, inflammation, which may decrease the ability to exercise and adhere to a training program [2]. Additionally, intense exercise is often followed by a period of weakness and soreness that can last for days or weeks with peak generally happening between 24 and 48h following exercise [3]. Olympic weightlifting exercises, well known as a very intense common type of strength training, are among the exercises concerned by these problems [4, 5]. In this context, and according to Lui et al. [4] and Pettersson. [5], both long-term exercise training and one week of intensive resistance training increased oxidative stress, muscle damage and cell injury in elite weightlifters. Therefore, appropriate rest after intensive training was found to be important for recovery. Recently, Ammar et al. [6, 7] demonstrated that markers of muscle damage, oxidative stress and inflammation increased immediately after an Olympic-weightlifting session with higher rates of increase in the morning compared to the evening and afternoon sessions. They have shown also that 48h are not enough to recover the rest values for the majorities of these parameters (i.e., especially after the morning session) [6, 7].

On the other hand, accumulating data clearly claimed that pomegranate, described as the new “super fruit” [8], has several health benefits. Pomegranates can help prevent or treat various disease risk factors including high blood pressure, high cholesterol, oxidative stress, hyperglycemia, and inflammatory activities [9, 10]. In fact, supplementation in pomegranate juice (POMj), containing high levels of polyphenols, was shown to: (i) reduce free radicals, oxidative stress and lipid peroxidation (-65%) [11], (ii) reduce risk of cardiovascular diseases [9, 12] by reducing high systolic blood pressure (-12%), carotid artery thickness (-30%), low-density lipoprotein cholesterol (LDL) oxidation (-90%), and by enhancing myocardial blood flow (+17%) [13] and antioxidant status (+130%) [9], and (ii) promote inhibition of some cellular transcription factors such as the nuclear factor NF-κB (NF-κB), tumor necrosis factor α (TNFα) and cyclooxygenase-2 (COX -2) [8, 14, 15], block their production and combat inflammatory degeneration of cartilage to protect articulations [16]. Additionally, in a spectrophotometric comparative study between POMj, red wine, blueberry juice, cranberry juice, orange juice and green tea [17], POMj was found to have the highest capacity to destroy free radicals and to reduce LDL oxidation and inhibit cellular oxidative stress in macrophages, with an antioxidant activity (Trolox equivalent antioxidant capacity: TEAC = 18–20) three times higher than red wine and green tea (6–8 TEAC) [17]. The efficacy of POMj is mainly due to its high bioavailability compared to other polyphenols such as resveratrol [18]. The underlying mechanisms of the biological polyphenol benefits are not yet clear but it was clearly proved especially in people placed under stressful situations [9,14,16,19]. In this context, despite the numerous health benefits of POMj in people under stress and despite that intensive physical exercise is one of the most stressed situations characterized by acute and delayed alteration in organism’s responses, few studies associated the effect of the pomegranate with the physical exercise [3, 20]. These studies mainly focused on potential effect of dietary supplements in POMj on muscle recovery from a bout of intense eccentric exercise [3, 20]. In untrained subjects, Trombold et al. [20] showed that a daily drink of 500ml POMj for five days prior to damaging eccentric exercise significantly improves the strength recovery 2–3 days after the exercise. However, these authors failed to find a significant effect upon the levels of muscle damage (creatine kinase (CK) and myoglobin) and inflammatory (interleukin-6, and CRP) markers during recovery. In another study conducted in trained participants (i.e. weight trained), it has been found that 15 days of drinking POMj (i.e., twice daily of 250ml) improve the arm strength during the 2 to 168-hour period post exercise and reduce significantly the elbow flexor muscle soreness for 48 and 72 hours post exercise [3]. However, isometric strength and muscle soreness in the knee extensors were not significantly affected by POMj supplementation compared to PLA. These authors conclude that POMj supplementation attenuates weakness and reduces soreness of the arm muscle but not leg muscles. Recently, Machin et al [21], searching for an optimal dose of pomegranate supplementation, showed that either once-daily or twice-daily dietary pomegranate juice supplementation improves strength recovery of both leg and arm muscles after an unaccustomed eccentric exercise. To the best of the authors’ knowledge, it seems to be no studies that examined the acute and delayed effects of POMj supplementation on performance and recovery after exercises involving whole body muscles (i.e. Olympic weightlifting exercises). Given the lack of research in this field and the contradictory results, the purposes of the present study were to check if natural POMj supplementation during the morning training session and 48h before could thwart the decline in the morning weightlifting performance and reduce the acute and delayed biochemical and muscle soreness responses.

## Methods

The authors confirm that all ongoing and related trials for this intervention are registered.

### Participants

Nine male elite weightlifters (age: 21 ± 0.5 years, body mass: 80 ± 9.5 kg, height 175 ± 8.1 cm (mean ± SD)) volunteered to participate in this study. The participants were recruited on the basis of: (i) they trained at least five sessions per week (between 15h:30 and 17h:30) with 90 to 120 min per session,(ii) they had an experience of more than 3 years in Olympic weightlifting and (iii) they didn’t have any injuries and they didn’t use any antioxidant (e.g., vitamin E, A, C etc.) or anti-inflammatory drugs during the experimental period and one month before. After receiving a thorough explanation of the possible risks and discomforts associated to the experimental procedures, each participant provided a written informed consent to take part in the experiment. The study was conducted according to the Declaration of Helsinki. The protocol (S1 Protocol) and the consent form were fully approved by the review board “Local committee of the Laboratory of Biochemistry, CHU Habib Bourguiba, Sfax, Tunisia” before the commencement of the assessments.

### Experimental design

**Fig 1:**
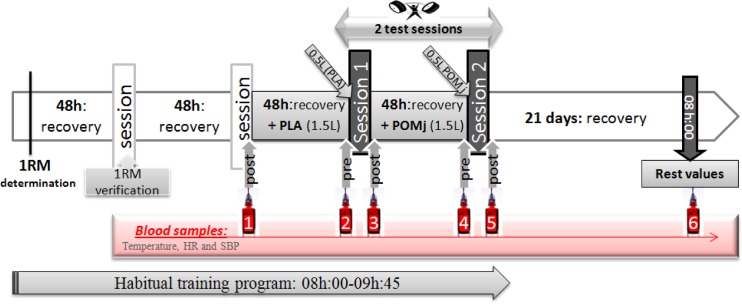
Experimental design. *NB*: the pre sessions 1 and 2 values correspond to values of 48-h recovery; Weightlifting performance was calculated during both test sessions; Acute (RPE) and delayed (DOMS) perceived muscle fatigue and soreness were assessed respectively 3min and 48 after both test sessions.

One week before the start of the experimental period (01/01/2015), the heaviest weight lifted in a single repetition (1-RM) was assessed for each participant in each Olympic movement Fig 1. After an ascending warm-up (i.e., 1^st^ set with 8 to 10 repetitions at 50%, 2^nd^ set with 3 to 5 repetitions at 75% and 3^rd^ set with 1 to 3 repetitions at 90% of athlete's estimated maximum), 1-RM was assessed in three trials with 5 min recovery time in-between [22]. As suggested by Kraemer and Fry [23], on-going encouragement and communication with the athletes during this testing is crucial to obtain the best performance. Also, estimated 1-RM was verified in the next two days. Then participants performed-as part of their habitual training program from 08h:00 to 09h:45- two training sessions Fig 1 using respectively, PLA (07/01/2015) and POMj (09/01/2015) supplementations with a recovery period of 48 h in between. Supplements (1500 ml) of PLA or POMj were taken three times daily in the 48h that proceeded respectively these two training sessions (i.e. 250ml × 6 times with 8-h intervals between it). Moreover, 1h before the training sessions, participants consumed an additional 500 ml of PLA and 500 ml of POMj, respectively Fig 1. Upon arrival for their first test session, each participant's body mass (Tanita, Tokyo, Japan) and height were recorded. Moreover, before and after each training session, oral temperature was recorded with a calibrated digital clinical thermometer (Omron, Paris, France; accuracy: 0.05°C) inserted sublingually for at least 3 min with the participant in a seated resting position for at least 15 min. In addition, before and after each training session, fasting blood samples (blood sample 2, 3, 4 and 5, Fig 1) were collected and heart rate (HR) and systolic blood pressure (SBP) were recorded using a heart rate monitor and a manual sphygmomanometer. Additionally, to assess the recovery kinetic of the biological parameters, blood sample, temperature, HR and SBP were collected at resting state (i.e., after 10 days of recovery, blood sample 6) and immediately (3min) after the training session which preceded the PLA session (blood sample 1). RPE was recorded after each training session [24]. Before test session, participants were fasting and allowed to drink only one glass of water (15–20 cl) to avoid the effects of postprandial thermogenesis [25]. Based on the results of Petterson et al. [5] who showed that biomarkers of muscle damage remain raised for at least 7days following intensive weightlifting exercises, a recovery period of 3weeks was chosen to evaluate the biological resting state (19/01/2015). Moreover, Given that (i) previous reports postulated that a minimal period for supplementation is at least (e.g., 30–60 minutes) before the workout [26] and (ii) POMj is a highly concentrated source of dietary nitrate and polyphenols [27], participants were asked to consume the 500 ml of POMj and PLA 60 minutes prior to the beginning of the correspondent training session to allow sufficient time for circulating nitrate and nitrite to become elevated to get an observable ergogenic effect. Additionally, given that (i) using randomized order in this study will results in consuming POMj then PLA supplementations for some participants with only 48h wash-out period, while (ii) the delayed beneficial effects of POMj could persist up to 3weeks after the interruption of consumption [28], authors in the present study tried to avoid any alteration in the biochemical blood levels at the beginning and during PLA administration period, by avoiding the consumption of POMj before PLA. Therefore, PLA (at first) then POMj was administrated for all participants together (non-randomized design).

### Training session

Each training session includes three Olympic-Weightlifting exercises: snatch, clean and jerk, and squat [6, 7, 29]. After increasing the load until unsuccessful trial in the snatch and the clean and jerk, five sets for each exercise were performed (i.e., two sets at 85% of 1-RM with three repetitions per set and three sets at 90% of 1- RM with two reps per set) with a passive recovery period of 5 min in-between [30]. Each session was preceded by 10 min warm-up wherein the participants will perform 3–5 repetitions with increasing loads (i.e., from 40 to 80% of the 1-RM). Performance in each session was measured by the total volume lifted in the two Olympic movements (volume lifted (kg) in snatch added volume lifted (kg) in clean and Jerk: only the right lifts are taken into consideration) [6, 7] and by the maximal lifted amounts in both Olympic movement (i.e., the maximal load lifted (kg) in the snatch added to the maximal load lifted (kg) in the clean and Jerk).

### Rating of perceived exertion scale

The rating of perceived exertion (RPE) scale (15-points) runs from 6 (very, very light) to 20 (very, very hard) 10. RPE scale measures feelings of effort, strain, discomfort, and/or fatigue experienced during physical task. Although this is a subjective measure (person's exertion), RPE values were shown to provide good estimation of the increase in heart rate, muscle fatigue and several other physiological measures during physical activity [24].

### Delayed onset of muscle soreness

Delayed onset soreness (DOMS) of the knee extensors and elbow flexors was determined 48 hours after the training sessions. Participants were asked to rate subjectively the degree of soreness in both muscles using a visual analog scale of 0 (absence of soreness) to 10 (unbearable soreness) [31]. Soreness was normalized to 100% of the maximal perceived level.

### Dietary records

To assess the adequacy of nutrient intake, a consecutive dietary record over 7 days was completed. All participants received a detailed verbal explanation and written instructions on data collection procedures. Participants were asked to continue with their usual dietary habits during the period of dietary recording and to be as accurate as possible in recording the amounts and types of food and fluid consumed. A list of common household measures, such as cups and tablespoons, and specific information about the quantity in each measurement (grams, etc.) were given to each participant. Each individual's diet was calculated using the Bilnut 4 software package (SCDA Nutrisoft, Cerelles, France) and the food composition tables published by the Tunisian National Institute of Statistics in 1978. Estimated nutrient intakes were referred to reference dietary intakes for physically active people and the daily nutriment data showed that total calorie, macronutrient, and micronutrient intakes are situated in the interval of the reference dietary intakes for healthy Tunisian adults.

### Pomegranate and placebo supplementations

The tested quantity of the natural POMj was prepared from a fresh pomegranate fruit 48h before the beginning of the experimentation and was frozen and stored at -4°C. No additional chemical products were added to the natural POMj. Each 500-mL of the tested POMj contained 2.56g of total polyphenol, 1.08g of orthodiphenols, 292.59mg of flavonoids and 46.75mg of flavonols. Subjects were reminded verbally through phone communication to consume at the required times their supplements. PLA juice consisted of a Pomegranate-flavored commercial drink contained water, citric acid, natural flavor and natural identical flavor (Pomegranate), sweeteners (aspartame × (0.3g/l), acesulfame K (0.16g/l)), stabilizers (Arabic gum) and didn’t contain antioxidants, vitamins nor polyphenols.

Given that the daily drink of 500ml (2 × 250ml/day) POMj for five or fifteen days prior to an intense exercise showed contradictory effects in the recovery levels of muscle damage, as well as muscle strength and soreness [3, 20], and given that the present study investigate the effect of the natural POMj consumed only 2 days before the training session, a supplementation quantity of 750ml (3 × 250ml /day) has been chosen.

### Phenolic compounds

#### Extraction of phenolic fraction

The phenolic extracts were obtained following the procedure of Chtourou et al. [32] with some modifications. In fact, the POMj sample (4g) was added to 2 mL of *n*-hexane and 4 mL of a methanol/water (60:40, v/v) mixture in a 20 mL centrifuge tube. After vigorous mixing, they were centrifuged for 3 min. The hydroalcoholic phase was collected, and the hexane phase was re-extracted twice with 4 mL of the methanol/water (60:40, v/v) solution each time. Finally, the hydro alcoholic fractions were combined, washed with 4 mL of *n*-hexane to remove the residual POMj, then concentrated and dried by evaporative centrifuge in vacuum at 35°C.

#### Determination of the total phenols and o-diphenols contents

The determination of the total phenolic compounds was performed by means of the Folin-Ciocalteau reagent using the method described by Gargouri et al. [33]. The total phenolic content was expressed as milligrams of gallic acid (GA) equivalent per kilogram of POM (y = 0.011x, R² = 0.990). The optical density (OD) was measured at λ = 765 nm, using a spectrophotometer (Shimadzu UV-1800 PC, Japan). The concentration of *o*-diphenolic compounds in the methanolic extract was determined by the method of Dridi-Gargouri et al. [34]. The total *o*-diphenolic content was expressed as milligrams of GA equivalent per kilogram of POM (y = 1.144x, R² = 0.999). The OD was measured at λ = 370 nm, using the same spectrophotometer.

#### Determination of total flavonoids

Total flavonoids were measured by a colorimetric assay developed by Gargouri et al. [33]; 1 mL aliquot of appropriately diluted sample or standard solutions of catechin (20, 40, 60, 80 and 100 mg L-1) was added to a 10-mL volumetric flask containing 4 mL double-distillate H_2_O. At zero time, 0.30 mL 5% NaNO_2_ was added to the flask. After 5 min, 0.30 mL 10% AlCl_3_ was added. At 6 min, 2 mL (1 mol L-1) NaOH was added to the mixture. Immediately, the reaction flask was diluted to volume with the addition of 2.40 mL of double-distillate H_2_O and thoroughly mixed. Concerning the absorbance of the mixture, pink in color, it was determined at 510 nm versus prepared water blank. As for the total flavonoids of fruits, they were expressed on a fresh weight basis as mg 100 g-1 catechin equivalents. It is worth noting that the samples were analyzed in triplicate.

### Blood sampling and analysis

Blood samples (7ml) were collected six times for each participant Fig 1 from a forearm vein (i.e., 3.5ml in tube containing EDTA for hematological parameters and 3.5ml in tube containing Heparin for GLC, CRE, CRP and muscle damage parameters). Blood samples were collected after 5 min of being seated at rest (blood sample 6), and before the two test sessions (blood samples 2 and 4). Immediately after the training sessions (blood samples 1, 3 and 5), samples were also collected at 3min of being seated. Samples were placed in an ice bath and centrifuged immediately at 3000 rpm (× g) for 10 min. Aliquots of the resulting plasma were stored at -80°C until analysis.

To eliminate inter-assay variance, all samples were analyzed in the same assay run. All assays were performed in duplicate in the same laboratory with simultaneous use of a control serum from Randox. Hematological parameters (i.e., white blood cells (WBC), neutrophils (NEU), red blood cells (RBC), and platelets (PLT) were performed within 3h in a multichannel automated blood cell analyzer Beckman Coulter Gen system-2 (Coulter T540). Plasma glucose (GLC), Creatinine (CRE), muscle damage markers and CRP were determined spectrophotometrically using Architect Ci 4100 d'ABOTT. CK, Alkaline phosphate (PAL), lactate dehydrogenase (LDH) and CRP were respectively measured with N-acetyl-L-cysteine method; the hydrolyze of phosphate parametrophenyl method; the oxidation of lactate on pyruvate method and the immunoturbidimetric method. The intra-assay coefficients of variation for these parameters kit were respectively 1.3%, 2.3%, 0.2% and 1.16%. Aspartate aminotransferase (ASAT) was determined by measuring NADH oxidation with intra-assay coefficient of variation of 1.1.

### Statistical analyses

All statistical tests were processed using STATISTICA 10.0 Software (Stat-Soft, Maisons-Alfort, France). Normality of the distribution was confirmed using the Shapiro–Wilks W-test. Paired simple t-test was used to analyze the effect of POMj supplementation on physical performances and RPE. To analyze the effect of POMj supplementation on the biological responses during training sessions (pre-post values), a two-way ANOVA (2 levels [supplementation (PLA and POMj] × 2 levels [training (Pre and Post]) with repeated measures was used. To analyze the effect of POMj supplementation in the recovery kinetic of the selected parameters, one-way ANOVA was used. Post hoc tests were conducted when significant main effects were found using Tukey's honest significance difference (HSD) test. Effect sizes were calculated as partial eta-squared (η_p_^2^) for the ANOVA analysis and as Cohen's d for the paired sample t-test to assess the practical significance of the findings. Pearson correlation was used to assess the correlation between DOMS and CK values. Significance was set at p < 0.05.

## Results

### Effect of pomegranate supplementation on physical performance, rating of perceived exertion and delayed onset soreness

**Fig 2:**
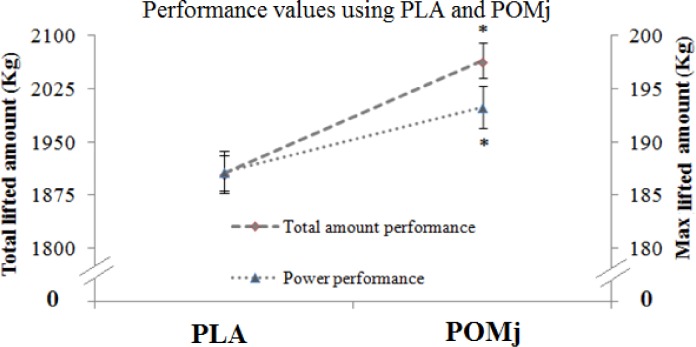
Calculated performances with PLA and natural POMj supplementations. *:Significant differences between PLA and POMj conditions.

**Fig 3:**
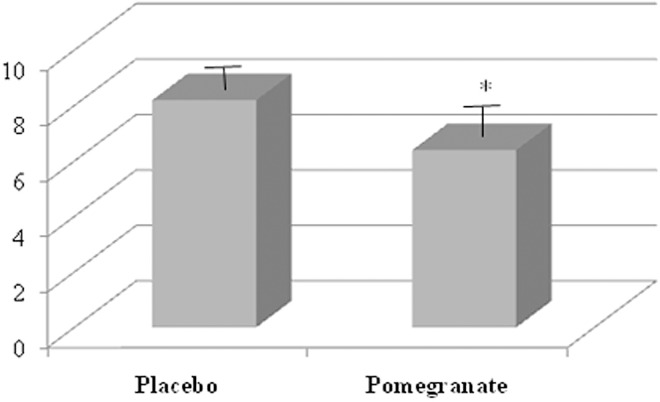
RPE values using Placebo and natural POMj supplementations. *: Significant differences between PLA and POMj conditions.

**Fig 4:**
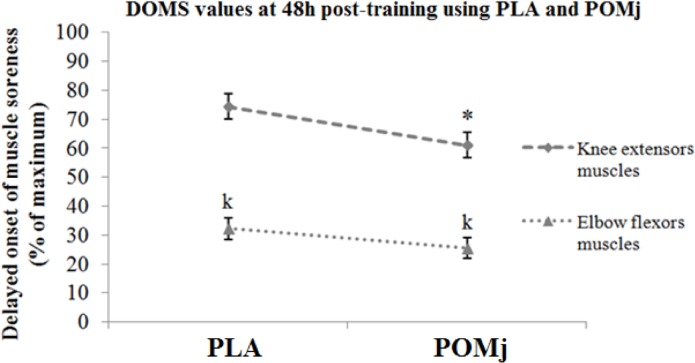
DOMS values using PLA and natural POMj supplementations. *: Significant differences between PLA and POMj conditions; k: Significant differences between DOMS of knee extensors and DOMS of elbow flexors.

Measurements of physical performance (i.e., Total lifted amount and maximal lifted amount) after using PLA and POMj supplementation were presented in Fig 2. Statistical analysis showed a significant POMj supplementation effect in both calculated performances (t = -2.37, p<0.05, d = -0.3 and t = -2.4, p<0.05, d = -0.1, respectively) during POMj condition compared to PLA (i.e., rate of increase = +8.29±3.8% and +3.26±0.83%, respectively for the total and the maximal lifted amounts). Similarly, RPE Fig 3 and DOMS (i.e., knee extensors; Fig 4) values showed significant effects of supplement (t = 2.4, p<0.05, d = 0.18 and t = 4, p<0.01, d = 0.42, respectively) with lower values being measured during POMj condition (i.e., rate of decrease = -4.37±1.45% for RPE and -13.4±3.84% for the knee extensors’ DOMS).

However, for the DOMS of the elbow flexors muscles Fig 4, no POMj effect was shown (p>0.05). In both conditions, delayed soreness of the knee extensors was higher (p<0.001) than delayed soreness of elbow flexors (i.e., 74.4±4.4% vs 32.2±3.6% and 61.1±5.1% vs 25.5±3.7%). For both muscle groups, significant correlations between the delayed soreness and the CK levels were shown (p<0.001) with r = 0.8 between the CK level and the DOMS of the knee extensors and r = 0.7 between the CK level and the DOMS of the elbow flexors.

### Effect of pomegranate on the biological parameters before and after the training sessions

The biological responses during the weightlifting training sessions were presented in Table 1 for both PLA and POMj supplementation.

**Table 1:** Acute biological responses to weightlifting training sessions using natural POMj and PLA supplementations.

Variables	Placebo			Pomegranate			Δ (POMj-PLA) in %	ANOVA		
	Pre	Post	% of change	Pre	Post	% of change		Pomegranate effect	Training effect	Interaction
**Temperature** (°C)	36.14±0.69	36.98±0.51 ^*^	+02.32%	36.06±0.82	37.03±0.48 ^*^	+02.74%	+00.42%	F = 0.1, p>0.05, ηp^2^ = 0.01	***F = 22*.*8*, *p<0*.*01*, *ηp***^***2***^ ***= 0*.*7***	F = 2.1, p>0,05, ηp^2^ = 0.2
**HR** (bpm)	72.44±9.00	85.11±10.6 ^*^	+18.22%	70.78±9.64	79.78±7.93 ^*^	+13.76%	-04.46%	***F = 9*.*6*, *p<0*.*05*, *ηp***^***2***^ ***= 0*.*5***	***F = 18*.*6*, *p<0*.*05*, *ηp***^***2***^ ***= 0*.*6***	F = 4.1, p>0.05, ηp^2^ = 0.3
**SBP** (mmHg)	12.49±0.96	13.67±0.35 ^*^	+09.89%	11.59±0.37 ^**P**^	12.52±0.72 ^*^	+8.08%	-01.81%	***F = 13*.*7*, *p<0*.*01*, *ηp***^***2***^ ***= 0*.*6***	***F = 26*.*7*, *p<0*.*001*, *ηp***^***2***^ ***= 0*.*7***	F = 1.1, p>0.05, ηp^2^ = 0.1
**GLC** (mmol/l)	05.02±0.50	05.05±0.59	NS	04.80±0.41	04.30±0.67 ^*^	-10.59%	———	***F = 9*.*7*, *p<0*.*05*, *ηp***^***2***^ ***= 0*.*5***	F = 3.1, p>0.05, ηp^2^ = 0.3	F = 1.1, p>0.05, ηp^2^ = 0.1
**CRE** (μmol/l)	82.00±8.26	83.67±9.43	NS	82.11±8.75	87.33±10.3 ^*^	+6.32%	———	F = 1.45, p>0.05, ηp2 = 0.1	***F = 14*.*67*, *p<0*.*01*, *ηp2 = 0*.*7***	F = 5.2, p>0.05, ηp^2^ = 0.2
**WBC** (10^3^ /μl)	08.62±2.67	07.52±2.03 ^*^	-11.36%	08.36±2.19	07.39±1.85	NS	———	F = 1.1, p>0.05, ηp^2^ = 0.1	***F = 12*.*9*, *p<0*.*01*, *ηp***^***2***^ ***= 0*.*6***	F = 0.1, p>0.05, ηp^2^ = 0.01
**NEU** (10^3^ / μl)	05.34±2.12	04.87±1.86 ^*^	-07.97%	04.83±1.90 ^**P**^	04.68±1.92	NS	———	***F = 9*.*9*, *p<0*.*05*, *ηp***^***2***^ ***= 0*.*5***	F = 3.8, p>0.05, ηp^2^ = 0.3	F = 1.4, p>0.05, ηp^2^ = 0.1
**RBC** (10^6^ / μl)	06.08±0.54	05.43±0.41 ^*^	-10.34%	05.51±0.59	05.55±0.24	NS	———	F = 2.14, p>0.05, ηp2 = 0.1	***F = 13*.*3*, *p<0*.*01*, *ηp***^***2***^ ***= 0*.*5***	***F = 7*.*1*, *p<0*.*05*, *ηp***^***2***^ ***= 0*.*6***
**PLT** (10^3^ / μl)	162.1±38.2	200.3±42.9 ^*^	+24.97%	184.1±43.6 ^**P**^	199.8±48.7	NS	———	F = 1.49, p>0.05, ηp2 = 0.1	***F = 14*.*1*, *p<0*.*01*, *ηp***^***2***^ ***= 0*.*7***	F = 1.2, p>0.05, ηp^2^ = 0.1
**CK** (UI/L)	342.4±42.2	440.3±56.2 ^*^	+28.28%	262.1±19.6 ^**P**^	311.8±21.4 ^*^	+19.53%	-8.75%	***F = 9*. *3*, *p<0*.*05*, *ηp***^***2***^ ***= 0*.*5***	***F = 42*.*2*, *p<0*.*001*, *ηp***^***2***^ ***= 0*.*7***	***F = 9*.*8*, *p<0*.*05*, *ηp***^***2***^ ***= 0*.*5***
**LDH** (U/l)	169.7±24.9	198.3±43.9 ^*^	+16.26%	156.0±23.3 ^**P**^	178.6±25.4 ^*^	+14.61%	-1.64%	***F = 5*.*7*, *p<0*.*05*, *ηp***^***2***^ ***= 0*.*4***	***F = 31*,*1*, *p<0*.*001*, *ηp***^***2***^ ***= 0*.*7***	F = 0.06, p>0.05, ηp^2^ = 0.2
**ASAT** (UI/l)	26.11±7.61	30.44±9.15 ^*^	+16.59%	23.33±5.29	26.22±4.14	NS	———	***F = 5*.*9*, *p<0*.*05*, *ηp***^***2***^ ***= 0*.*4***	***F = 37*.*9*, *p<0*.*001*, *ηp***^***2***^ ***= 0*.*6***	F = 0.01, p>0.05, ηp^2^ = 0.01
**PAL** (UI/l)	72.44±13.5	75.67±13.8 ^*^	+04.51%	72.78±16.4	72.67±14.3	NS	———	F = 0.7, p>0.5, ηp^2^ = 0.1	***F = 6*.*4*, *p<0*.*05*, *ηp***^***2***^ ***= 0*.*4***	F = 3.1, p>0.05, ηp^2^ = 0.3
**CRP** (mg/l)	01.39±0.91	01.48±0.91 ^*^	+12.59%	01.30±0.88	01.32±0.89	NS	———	F = 2.6, p>0.05, ηp^2^ = 0.01	F = 5.1, p>0.05, ηp^2^ = 0.4	F = 2.5, p>0.05, ηp^2^ = 0.14

Temperature, Heart rate (HR), Systolic blood pressure (SBP), Blood Glucose (GLC), Creatinine (CRE), White blood cells (WBC), Neutrophils (NEU), Red blood cells (RBC), Platelets (PLT), Creatinine kinase (CK), Lactate dehydrogenase (LDH), Aspartate aminotransferase (ASAT), Alkaline phosphate (PAL) and C-reactive protein (CRP) represented as mean ± $S_{\bar{x}}$ at pre and post training sessions using POMj and PLA supplementations.

^*^ Significant differences between pre-post training sessions.

^P^ Significant differences between POMj and PLA values at pre training sessions (i.e., 48h recovery).

#### Effect of POMj on core temperature, heart rate systolic blood pressure, glucose and creatinine

**Acute effect:** Statistical analysis showed a significant increase pre-post training session for core temperature, HR and SBP in both conditions (p<0.001) with higher rate of increase for core temperature (+0.42%) and lower rate of increase for HR and SBP (-4.46% and -1.81%) during POMj condition. There is also a significant difference pre-post training session for GLC and CRE only using POMj with lower post training values for GLC (p<0.05, rate of decrease = -10.59±3.51%) and higher one for the CRE (p<0.05, rate of increase = +6.32±1.57%).

**Delayed effect:** Additionally, a significant difference in pre-training sessions values (i.e., after 48h recovery using 1.5L of PLA vs. 1.5L of POMj supplementations) was recorded for SBP with lower values using POMj supplementation (p<0.01).

For these parameters, a significant effect of POMj supplementation were recorded for HR and GLC with p<0.05 and for SBP with p<0.01 and significant effects of training session were recorded for core temperature, HR, SBP, and CRE (p<0.01, p<0.05, p<0.001 and p<0.01, respectively) while no interaction POMj supplementation × Training session was registered.

#### Effect of POMj on hematological parameters

**Acute effect:** Concerning the hematological parameters, during the PLA condition, all the parameters showed a significant difference pre-post training session with lower post-training values for WBC, NEU and RBC (p<0.05, rate of decrease = -11.36±3.66%, -7.97 ±4.11% and -10.34±2.02%, respectively) and higher post-training values for PLT (p<0.01; rate of increase = +24.97±9.95%). Using POMj supplementation, no significant change pre-post session was registered (p>0.05) for all the selected parameters.

**Delayed effects:** The comparison of the pre-training values in both conditions, shows a significant difference for NEU and PLT with lower pre-training values for NEU (p<0.05) and higher ones for PLT (p<0.05) using POMj supplementation.

For these parameters, significant POMj effects were registered for NEU (p<0.05), significant training effects were registered for WBC, RBC and PLT (p<0.001) and a significant interaction POMj supplementation × Training session was registered for RBC (p<0.05).

#### Effect of pomegranate on muscle damage parameters and C—reactive protein

**Acute effects:** For the muscle damage and inflammatory markers, during both PLA and POMj conditions, significant increases pre-post training sessions were registered for CK and LDH (p<0.001 and p<0.01) with lower rate of increase (-8.75% and -1.64%) using POMj supplementation. However, ASAT, PAL and CRP showed a significant difference (p<0.05) pre-post training session only with PLA condition with higher values at post-training session (rate of increase = +16.59%, +04.51% and +12.59% respectively).

**Delayed effects:**The comparison of the pre-training values in both conditions, showed a significant difference only for CK (p<0.001) and LDH (p<0.05) with lower values using POMj.

Similarly, significant POMj supplementation (p<0.05) and training session effect (p<0.001) were registered for these three markers (i.e., CK, LDH and ASAT). However, for PAL and CRP, only significant training effects (p<0.05) was registered for PAL. A significant interaction POMj supplementation × Training session was registered only for CK (p<0.05).

#### Recovery kinetic of the biological parameters using placebo and pomegranate supplementations

Tables 2 and 3 show the values of biological parameters at resting state and immediately and 48 after the training sessions during respectively PLA and POMj conditions.

**Table 2:** Effect of 48h of recovery period associated to PLA supplementation in the biological responses.

Variables	Placebo			Δ 48h-3’ in %	ANOVA
	3’Post training	48h recovery	Rest values		
**Temperature** (°C)	37.03±0.45	36.14±0.69^**A**^	36.18±0.69	-02.39%	***F = 14*.*6*, *p<0*.*001*, *ηp***^***2***^ ***= 0*.*4***
**HR** (bpm)	86.56±8.60	72.44±9.00^**A**^	69.78±9.74	-16.04%	***F = 30*.*2*, *p<0*.*001*, *ηp***^***2***^ ***= 0*.*4***
**SBP** (mmHg)	13.44±0.56	12.49±0.96^**A**^	12.17±0.90	-07.19%	***F = 37*.*0*, *p<0*.*001*, *ηp***^***2***^ ***= 0*.*5***
**GLC** (mmol/l)	05.08± 0.59	05.02±0.50	04.88±0.48	NS	F = 0.74, p>0.05, ηp2 = 0.1
**CRE** (μmol/l)	83.56±8.23	82.00±8.26	83.44±9.18	NS	F = 2.98, p>0.05, ηp2 = 0.1
**WBC** (10^3^ /μl)	07.77±2.11	08.62±2.67^**A**^	08.17±2.25	+10.82%	***F = 3*.*67*, *p<0*.*05*, *ηp***^***2***^ ***= 0*. *3***
**NEU** (10^3^ /μl)	04.99±1.41	05.34±2.12	05.19±1.68	NS	F = 0.71, p>0.05, ηp2 = 0.1
**RBC** (10^6^ / μl)	05.90±0.62	06.08±0.54	05.73±0.63	NS	F = 1.99, p>0.05, ηp2 = 0.1
**PLT** (10^3^ /μl)	196.8±37.1	162.11±38.2^**A**^	181.9±46.0	-16.07%	***F = 4*.*16*, *p<0*.*05*, *ηp2 = 0*.*4***
**CK** (UI/L)	451.2±55.8	342.4±42.2^**A**^^**B**^	237.4±36.5	-23.98%	***F = 29*.*3*, *p<0*.*001*, *ηp***^***2***^ ***= 0*.*5***
**LDH** (U/l)	197.2±40.1	169.7±24.9^**A**^^**B**^	145.3±18.2	-12.97%	***F = 37*.*8*, *p<0*.*001*, *ηp***^***2***^ ***= 0*.*3***
**ASAT** (UI/L)	30.77±7.59	26.11±7.61^**A**^^**B**^	20.11±3.82	-14.70%	***F = 16*.*3*, *p<0*.*001*, *ηp***^***2***^ ***= 0*.*4***
**PAL** (UI/L)	70.67±12.9	72.44±13.5	73.22±16.0	NS	F = 2.16, p>0.05, ηp2 = 0.2
**CRP** (mg/l)	01.46±0.90	01.39±0.91	01.24±0.70	NS	F = 2.87, p>0.05, ηp2 = 0.7

Temperature, Heart rate (HR), Systolic blood pressure (SBP), Blood Glucose (GLC), Creatinine (CRE), White blood cells (WBC), Neutrophils (NEU), Red blood cells (RBC), Platelets (PLT), Creatinine kinase (CK), Lactate dehydrogenase (LDH), Aspartate aminotransferase (ASAT), Alkaline phosphate (PAL) and C-reactive protein (CRP) represented as mean ± $S_{\bar{x}}$ at rest, 3min and 48h post training session using PLA supplementations.

^A^ Significant differences between 48h and 3min post training session.

^B^ Significant difference between 48h recovery and rest values. *NB: 3’ Post training correspond to blood sample 1, 48h recovery correspond to blood sample 2 and rest correspond to blood sample 6 Fig 1*

**Table 3:** Effect of 48h of recovery period associated to POMj supplementation in the biological responses.

Variables	Pomegranate			Δ 48h-3’ in %	ANOVA
	3’Post training	48h recovery	Rest values		
**Temperature** (°C)	36.98±0.51	36.06±0.82^**A**^	36.18±0.69	-02.49%	***F = 16*.*76*, *p<0*.*001*, *ηp***^***2***^ ***= 0*.*2***
**HR** (bpm)	85.11±10.6	70.78±9.64^**A**^	69.78±9.74	-16.60%	***F = 23*.*63*, *p<0*.*001*, *ηp***^***2***^ ***= 0*.*2***
**SBP** (mmHg)	13.67±0.35	11.59±0.37^**A**^	12.16±0.90	-15.16%	***F = 36*.*67*, *p<0*.*001*, *ηp***^***2***^ ***= 0*.*5***
**GLC** (mmol/l)	05.05±0.59	04.80±0.41	04.88±0.48	NS	F = 1.24, p>0.05, ηp2 = 0.1
**CRE** (μmol/l)	83.67±9.43	82.11±8.75	83.44±9.18	NS	F = 0.73, p>0.05, ηp2 = 0.1
**WBC** (10^3^ /μl)	07.52±2.03	08.36±2.19^**A**^	08.17±2.25	+11.42%	***F = 4*.*99*, *p<0*.*05*, *ηp***^***2***^ ***= 0*.*6***
**NEU** (10^3^ /μl)	04.87±1.86	04.83±1.90	05.19±1.68	NS	F = 2.67, p>0.05, ηp2 = 0.1
**RBC** (10^6^ / μl)	05.43±0.41	05.51±0.59	05.73±0.63	NS	F = 1.85, p>0.05, ηp^2^ = 0.1
**PLT** (10^3^ /μl)	200.3±42. 9	184.1±43.6	181.9±46.0	NS	F = 1.68, p>0.05, ηp^2^ = 0.1
**CK** (UI/L)	440.3±56.2	262.1±19.6^**A**^	237.4±36.5	-35.32%	***F = 17*.*1 p<0*.*001*, *ηp***^***2***^ ***= 0*.*4***
**LDH** (U/l)	198.3±43.9	156.0±23.3^**A**^	145.3±18.2	-20.27%	***F = 19*.*8*, *p<0*.*001*, *ηp***^***2***^ ***= 0*.*4***
**ASAT** (UI/L)	30.44±9.33	23.33±5.29^**A**^	20.11±3.82	-21.47%	***F = 11*.*5*, *p<0*.*001*, *ηp***^***2***^ ***= 0*.*3***
**PAL** (UI/L)	75.76±14.1	72.78±16.4	73.22±16.0	NS	F = 2.81, p>0.05, ηp^2^ = 0.1
**CRP** (mg/l)	01.48±0.91	01.30±0.88	01.24±0.70	NS	F = 2.69, p>0.05, ηp^2^ = 0.1

Temperature, Heart rate (HR), Systolic blood pressure (SBP), Blood Glucose (GLC), Creatinine (CRE), White blood cells (WBC), Neutrophils (NEU), Red blood cells (RBC), Platelets (PLT), Creatinine kinase (CK), Lactate dehydrogenase (LDH), Aspartate aminotransferase (ASAT), Alkaline phosphate (PAL) and C-reactive protein (CRP) represented as mean ± $S_{\bar{x}}$ at rest, 3min and 48h post training session using PLA supplementations.

^A^ Significant differences between 48h and 3min post training session.; *NB: 3’ Post training correspond to blood sample 1, 48h recovery correspond to blood sample 2 and rest correspond to blood sample 6 Fig 1*

#### Recovery kinetic of core temperature, heart rate, systolic blood pressure, glucose and creatinine

In both conditions (i.e., PLA and POMj), statistical analysis showed a significant decrease (p<0.001) from 3min to 48h after training sessions for oral temperature, HR and SBP with higher rate of decrease for SBP during POMj condition (7.97±1.52%). However, for GLC and CRE no significant changes were registered during the two conditions. For all these parameters (i.e., temperature, HR, SBP, GLC and CRE) and in both conditions, 48h of recovery was a sufficient period to return the post-training values (3min) to the resting levels.

#### Recovery kinetic of hematological parameters

Concerning the hematological parameters, WBC values showed a significant difference between 3min (post training) and 48h recovery values in both conditions with higher values after 48h (p<0.05, rate of increase = +10.82±2.99% using PLA and rate of increase = +11.42±3.68% using POMj). Moreover, there is a significant decrease of PLT between 3min (post training) and 48h recovery values only during PLA condition with lower values after the 48h recovery period (p<0.05, rate of decrease = -16.07±2.65%). However, for NEU and RBC no significant differences were observed during recovery in both conditions. For all the hematological parameters, 48h of recovery period was sufficient in both conditions to return the post-training values (3min) to the rest levels.

### Recovery kinetic of muscle damage parameters and c-reactive proteinCRP

For the muscle damage and inflammatory parameters, significant decreases from 3min to 48h after training session were registered during both PLA and POMj conditions for CK (p<0.01 and p<0.001), LDH (p<0.01 and p<0.001) and ASAT (p<0.05 and p<0.01) with higher rates of decreases (11.34±1.98%, 7.30±0.86% and 6.77±0.47%) using POMj. For these parameters, the 48h period was sufficient to recover the resting values only using POMj supplementation. In fact, during PLA condition, these parameters showed a significant difference between 48h recovery and rest values (p<0.01 for CK, ASAT and LDH). For PAL and CRP, no significant alterations were registered in both conditions.

## Discussion

The aim of this study was to investigate the effect of natural POMj supplementation on weightlifting performance, perception of muscle fatigue and soreness as well as the acute and delayed biochemical responses following a morning weightlifting training session. The main results showed that, comparing to PLA, the consumption of POMj 48h before and during the training session (i) ameliorated the total (+8.3%) and the maximal (+3.26%) lifted amount in the two Olympic movements and (ii) improved the perception of muscle fatigue (4.37%) immediately and the perception of the knee extensors muscle soreness (13.4%) 48h after the training session. Moreover, POMj modified the kinetic of the acute and delayed biological responses with a reduction in the immediate increase of the HR (-4.46%), SBP (-1.81%), CK (-8.75%) and LDH (-1.64%), an absence of significant increase of ASAT, PAL and CRP three min after the training session and an improvement in the delayed recovery kinetic of SBP, CK, LDH and ASAT (7.97%, 11.34%, 7.30% and 6.77%, respectively).

### Effect of pomegranate supplementation on the calculated performances

The total and the maximal amount raised in the two Olympic lifts (i.e. the Snatch and Clean& Jerk) during the training session were higher in POMj condition compared to PLA. These results suggest that POMj supplementation during the weightlifting training session and during the 48h that proceeds, improve the whole body muscle strength and ameliorate the capacity to adhere to intense training program. This study confirms the results of Trombold et al. [20], which showed that a daily drink of 0.5L POMj for five days prior to intense exercise improved the strength recovery 2–3 days after damaging eccentric exercise. In this sense, Machin et al. [21] showed that both once-daily (650 mg/d) and twice daily (1,300 mg /d) dietary polyphenol supplementation improves strength recovery of both leg and arm muscles after unaccustomed eccentric exercise. Similarly, Trombold et al. [3] have found that POMj ingested before the eccentric exercise and through the recovery period improved the arm strength (i.e., immediately (8–10%) and during the 2h to 168h period post exercise) and have no effect on the knee isometric strength. The present study results confirms these previous findings upon the potential effects of POMj on strength performances of the whole body muscles, as Olympic weightlifting exercises involve the majorities of human muscles [35].

### Effect of pomegranate on the acute and delayed muscle fatigue and soreness

In this study, significant effects of POMj supplementation were shown for RPE and for the DOMS of the knee extensors with lower values immediately and 48h after the corresponding training session. The present results of RPE were in line with the results of Trombold et al. [3, 20] showing that, in both untrained and trained subjects, a daily drink of POMj prior to intense eccentric exercise and during recovery improves the muscle soreness at 120 min after exercise. The lower levels of muscle fatigue and soreness felt immediately after exercises when using POMj (i) could be explained by a lower level of tissue oedema and/or a lower accumulation of metabolic by-products [36] and (ii) indicates that during the corresponding exercises, athletes felt less fatigable, and this can explain their better performance [3]. Concerning the recovery of muscle soreness in the 48h following the training session, the present findings showed a significant improvement using the POMj only in the soreness of knee extensors muscles but not in the elbow flexors. These results are in contradiction with those of Trombold et al. [3] who showed that POMj aid in the recovery of soreness only in the arm muscles but not in the leg muscles. The present absence of significant POMj effects on the delayed soreness of the elbow flexors could be explained by the fact that in both PLA and POMj conditions, weightlifting training exercises induced more soreness (+40±3%) in the leg muscles compared to the arm ones. The presence of a significant effect of POMj on the DOMS of the knee extensors in the present study, which was absent in the study of Trombold et al. [3], could be explained by the higher dose of Ployphenol in the present POMj (i.e., natural POMj contains 2.56g/500ml while the commercialized one contains only 650 mg/480ml).

Comparing the present effects of natural POMj with previous nutritional interventions (vitamins, anti-inflammatory etc), it seems that POMj has a powerful effect in improving muscle performance and reducing DOMS. Nevertheless, using vitamin supplementations (C or E), showed no influence on soreness or strength performance [37, 38]. Similarly, non-steroidal anti-inflammatory drug (*NSAID*) supplementation shows the improvement of soreness but has no effect on strength levels [39]. Ingestion of combination of ascorbic acid, α-tocopherol, and selenium [40] or mixture of tocopherols, flavonoids (i.e., Hesperetin and quercetin), selenium or docosahexaenoate [41] have been reported to reduce oxidative stress after eccentric exercise and to attenuate systematic (CRP and interleukin6) inflammation markers, respectively. However, strength performance and DOMS were not recorded in both studies. Therefore, POMj could be an effective treatment to improve the recovery of muscles’ strength and reduce fatigue and weakness. On the other hand, it was suggested that delayed soreness is associated with the inflammatory and muscle damage responses [36]. The present study confirms this suggestion and shows a higher correlation between DOMS (i.e. leg extensors and elbow flexors) and CK values (r = 0.8 and r = 0.7, respectively).

### Effect of the weightlifting training session on the biological levels

The present results showed a significant training effect for the majorities of biological studied parameters (i.e., hematological, muscle damage and inflammatory) with higher values of temperature, HR, SBP, CK, LDH, ASAT at post-training session compared to pre-training session in both conditions (i.e., PLA and POMj). These findings are in line with previous studies of Lui et al. [4], Petterson et al. [5] and Ammar et al [6, 7] which showed a significant increase of muscle damage and inflammatory marker following weightlifting training sessions.

### Effect of pomegranate on the acute and delayed biological response

Compared to the PLA condition, natural POMj modifies the acute responses for the majority of the tested parameters. Indeed, during POMj condition, (i) the significant differences pre-post training session were suppressed for WBC, NEU, RBC, ASAT, PAL and CRP, and (ii) a reduction in the rate of increase (pre-post training session) were shown for HR, SBP and the muscle damage parameters (i.e., CK and LDH). Moreover, only using POMj, a significant increase in CRE and a significant decrease in GLC were shown, indicating a higher GLC consumption and CRE production during the corresponding session. The above-mentioned physiological modifications using POMj could be in the origin of the better physical performances and lower fatigue perception observed in the second test session. Concerning the delayed effect, the present results showed that the consumption of POMj during the 48h of recovery period, accelerate the recovery kinetic of SBP, CK and LDH and increase the PLT level. The lower SBP level registered using the POMj supplementation confirms the previous result of Aviram et al. [9] who showed a reduction of high systolic blood pressure by 12% after a daily drink of 500ml of POMj. The significant delayed increase of the PLT levels using POMj suggests that POMj supplementation could be also an effective treatment for patients who suffer from the thrombocytopenia disease (i.e., disorder in which there is an abnormally low amount of platelets sometimes associated with abnormal bleeding [42]). However, confirming this suggestion using patients with thrombocytopenia disease is necessary. Moreover, the acute increase in CRP level only during PLA condition confirms previous findings [8, 14, 15] indicating that fermented pomegranate polyphenols have an anti-inflammatory effect explained by an inhibition of some inflammatory markers such as NF-κ-B, TNFα and COX-2. However, the present findings are in contradiction with those of Trombold et al. [20] who showed that a daily drink of 500ml POMj prior exercise for 5 days has no effect on the levels of inflammation (i.e., interleukin-6 and CRP) and muscle damage (i.e., CK and myoglobin). These contradictory results could be explained by the training levels of the participants and the daily dose of POMj. In fact, Trombold et al. [20] investigated untrained subjects while the present participants were elite weightlifters. Moreover, the present study dose of POMj was 250ml higher than the one of Trombold et al. [20]. Thus, authors speculate that a 750ml (3 × 250ml) daily drink of POMj could be an optimal dose to have a significant anti-inflammatory effect. Additionally, the present effect of POMj–which consists in a potent antioxidant supplementation [8–10]—in reducing the acute and delayed muscle damage and inflammatory responses, suggests a strong relation between the muscle damage, inflammatory and oxidative process. This suggestion is in line with previous studies of (i) Lui et al. [4] who showed a significant correlation between the malondialdehyde (MDA) and the CK, WBC and NEU, (ii) Cudney et al. [43] who suggested that lipid peroxidation could be in the origin of hematological and muscle damage alteration, and (iii) Tidball et al. [44] who suggested a causal relation between inflammatory responses and generalized muscle damage after strenuous exercise.

### Effect of pomegranate on the recovery kinetic of the biological parameters

Concerning the effect of 48h recovery period associated to PLA or POMj supplementations, the present results showed that this period was sufficient to recover the baseline levels of HR, SBP, GLC, CRE, PAL, CRP and all the hematological parameters, whereas, CK, LDH and ASAT remained elevated. These results confirm previous results of Ammar et al. [6, 7] who showed that 48h recovery period decreases significantly the CRP values and the majority of muscle damages markers compared to post-training session; but was not sufficient to recover the resting values of CK, LDH and ASAT. Likewise, the present study shows that using POMj supplementation during the recovery period helps in returning the post-training session values of CK, LDH and ASAT to their baseline concentrations. These results were especially due to the higher rate of decrease (i.e., 3min to 48h post training session) following the consumption of POMj supplement compared to the PLA. Indeed, the results showed that the rates of decrease were 11.34%, 7.30% and 6.77% higher respectively for CK, LDH and ASAT, using POMj supplementation. Similarly, with the same condition, the CRP showed higher rate of decrease (5.1%) during the 48h period. The present acceleration in the recovery kinetic of CK, LDH, ASAT and CRP, affirms the above-mentioned suggestion that pomegranate could be a potent anti-inflammatory and anti-damage treatment. In this context, it has been also found that polyphenol possess a high biological activity during stressed or pathological situation (i.e., characterized by higher inflammatory and oxidative levels) [16] and have also cardio-, cancer, atherosclerosis and rheumatoid arthritis protective effects [9, 12, 13].

The efficacy of POMj could be attributed to its high content of polyphenols. Indeed, using POM Wonderful bottle (Los Angeles, CA, with total polyphenol content of 650 mg in each 480-ml bottle [20]), has no reported effect on muscle damage levels. However, in the present study, using natural POMj -that contains 2.56g of polyphenol in each 500ml- showed an acceleration of muscle damage recovery following intense weightlifting training session.

Furthermore, using similar training session at different times of the day, Ammar et al. [6, 7] showed that performance (i.e, total lifted amount) was lower in the morning compared to the evening (i.e., range of -8.30 ± 3.02%), with higher RPE (+4.78±1.67%) and muscle damage levels at this time of day (TOD). The present results showed that using POMj supplementation, the morning performance (i.e., total lifted amount) and RPE values were improved by 8.29±3.8% and 4.37±1.45%, respectively and the muscle damage levels were decreased. Therefore, the authors suggest that the POMj consumption through the recovery period and during morning training could counteract the lower morning performance and the higher fatigability at this TOD. However, more studies include training session in different TOD associated to POMj supplementation are necessary to confirm these suggestions.

### Methodological considerations

Previous double-blind-randomized study which investigate the effect of POMj consumption on strength recovery have shown that 14-days could be a sufficient “wash-out” period for POMj [20]. Indeed, in relation with strength recovery assessment, Trombold et al. [20] reported no significant differences between trial 1 PLA and trial 2 PLA and between trial 1 POM and trial 2 POM, when ordering effects are assessed dependent of the treatment. However, in another study, the antioxidant beneficial effects of the POMj have shown to persist up to 3 weeks after consumption was halted [28]. These findings speculate that beneficial effect of POMj on both oxidative stress and muscle damages could persist for up to 3 weeks. Based on the above results, the authors of the present study preferred to not randomize the treatment-with POMj as second treatment-in the study of anti-damaging effects of POMj following intensive exercise with short wash-out period (e.g., 48h, especially using people required to work intensely every 2–3 d). A limitation of this study was that, having the POMj trial always second might have biased the results. Indeed, it is possible that the first control bout (i.e., PLA) offered the participants some level of protection from DOMS during the second bout (i.e., POMj) [45]. However, given that this experimental design was not a separate training where the body will adapt in each session; the order effect seems to be minimal.

## Conclusion

The present study showed that consuming POMj supplementation–in 9 elites athletes- during weightlifting training session and during the 48h before (i), reduce the acuteness of the pain and delayed responses of the muscle damage and inflammation, and muscle soreness (i.e., knee flexor) (ii) accelerate the recovery kinetic of the biological parameters and (iii) improve the weightlifting performance. However, given the small sample size, further studies should verify these results using greater sample of athletes.

## Supporting Information

S1 Individual Data Points Individual data points. (DOCX)

S1 Protocol Study Protocol. (DOCX)

S1 Receipt of the Trial Registry Receipt of the trial registry ((ClinicalTrials.gov PRS). (PDF)

S1 TREND Checklist Trend Statement Checklist. (PDF)
