# Supplementary material for: Pomegranate Supplementation Accelerates Recovery of Muscle Damage and Soreness and Inflammatory Markers after a Weightlifting Training Session
Source: PLoS One. 2016 Oct 20;11(10):e0160305. doi: 10.1371/journal.pone.0160305 (PMC5072630; doi:10.1371/journal.pone.0160305)
Supplement: S1 Individual Data Points — (DOCX) [file pone.0160305.s001.docx]

| Temperature | | | | | | |
| --- | --- | --- | --- | --- | --- | --- |
| Blood Sample | 1 | 2 | 3 | 4 | 5 | 6 |
| S1 | 37,4 | 37,4 | 37,7 | 37,4 | 37,6 | 37,3 |
| S2 | 37,2 | 36,1 | 36,9 | 36,2 | 37,2 | 36,7 |
| S3 | 36,5 | 36,5 | 36,9 | 36,5 | 36,9 | 36,4 |
| S4 | 37,1 | 35,3 | 36,8 | 35,3 | 37,1 | 35,35 |
| S5 | 37,6 | 36,1 | 37,2 | 36,1 | 37,1 | 36,1 |
| S6 | 37,6 | 36,3 | 37,5 | 36,4 | 37,5 | 36,8 |
| S7 | 36,8 | 36,7 | 36,8 | 36,6 | 37 | 36,1 |
| S8 | 36,6 | 35,7 | 37,1 | 34,8 | 37 | 35,7 |
| S9 | 36,5 | 35,2 | 35,9 | 35,2 | 35,9 | 35,2 |
| mean | 37,03 | 36,14 | 36,98 | 36,06 | 37,03 | 36,18 |
| SD | 0,42 | 0,65 | 0,48 | 0,77 | 0,46 | 0,65 |
| median | 37,10 | 36,10 | 36,90 | 36,20 | 37,10 | 36,10 |
| Variance | 0,20 | 0,48 | 0,26 | 0,67 | 0,24 | 0,48 |

| HR | | | | | | |
| --- | --- | --- | --- | --- | --- | --- |
| Blood Sample | 1 | 2 | 3 | 4 | 5 | 6 |
| S1 | 99,00 | 86,00 | 110,00 | 86,00 | 97,00 | 84,00 |
| S2 | 89,00 | 70,00 | 83,00 | 65,00 | 73,00 | 65,00 |
| S3 | 93,00 | 78,00 | 91,00 | 75,00 | 77,00 | 75,00 |
| S4 | 88,00 | 82,00 | 84,00 | 82,00 | 84,00 | 81,00 |
| S5 | 95,00 | 66,00 | 85,00 | 65,00 | 83,00 | 66,00 |
| S6 | 73,00 | 66,00 | 74,00 | 60,00 | 73,00 | 60,00 |
| S7 | 85,00 | 71,00 | 76,00 | 68,00 | 71,00 | 76,00 |
| S8 | 79,00 | 57,00 | 82,00 | 59,00 | 81,00 | 55,00 |
| S9 | 78,00 | 76,00 | 81,00 | 77,00 | 79,00 | 66,00 |
| mean | 86,56 | 72,44 | 85,11 | 70,78 | 79,78 | 69,78 |
| SD | 8,60 | 9,00 | 10,56 | 9,64 | 7,93 | 9,74 |
| median | 88,00 | 71,00 | 83,00 | 68,00 | 79,00 | 66,00 |
| Variance | 74,03 | 81,03 | 111,61 | 92,94 | 62,94 | 94,94 |

| SBP | | | | | | | | | | | |  |
| --- | --- | --- | --- | --- | --- | --- | --- | --- | --- | --- | --- | --- |
| Blood Sample | | 1 | | 2 | | 3 | 4 | | 5 | 6 | |  |
| S1 | | 14,20 | | 12,90 | | 13,90 | 12,10 | | 13,00 | 12,30 | |  |
| S2 | | 13,30 | | 12,50 | | 13,50 | 11,90 | | 12,50 | 11,90 | |  |
| S3 | | 13,00 | | 12,40 | | 13,30 | 11,10 | | 11,20 | 12,10 | |  |
| S4 | | 13,50 | | 13,40 | | 13,70 | 11,30 | | 12,60 | 12,20 | |  |
| S5 | | 14,10 | | 13,30 | | 13,90 | 11,60 | | 11,40 | 12,30 | |  |
| S6 | | 13,40 | | 12,10 | | 13,90 | 11,70 | | 13,20 | 12,10 | |  |
| S7 | | 14,00 | | 13,70 | | 14,20 | 11,50 | | 12,80 | 12,20 | |  |
| S8 | | 12,90 | | 11,00 | | 13,10 | 12,00 | | 13,00 | 11,00 | |  |
| S9 | | 12,60 | | 11,10 | | 13,50 | 11,10 | | 13,00 | 11,10 | |  |
| mean | | 13,44 | | 12,49 | | 13,67 | 11,59 | | 12,52 | 11,91 | |  |
| SD | | 0,56 | | 0,96 | | 0,35 | 0,37 | | 0,73 | 0,50 | |  |
| median | | 13,40 | | 12,50 | | 13,70 | 11,60 | | 12,80 | 12,10 | |  |
| Variance | | 0,32 | | 0,93 | | 0,12 | 0,14 | | 0,53 | 0,25 | |  |
| CRE | | | | | | | | | | | | |
| Blood Sample | 1 | | 2 | | 3 | | | 4 | 5 | | 6 | |
| S1 | 81,00 | | 80,00 | | 84,00 | | | 82,00 | 84,00 | | 80,00 | |
| S2 | 96,00 | | 95,00 | | 102,00 | | | 92,00 | 98,00 | | 99,00 | |
| S3 | 74,00 | | 74,00 | | 74,00 | | | 76,00 | 77,00 | | 75,00 | |
| S4 | 84,00 | | 83,00 | | 82,00 | | | 80,00 | 93,00 | | 88,00 | |
| S5 | 83,00 | | 79,00 | | 82,00 | | | 78,00 | 83,00 | | 81,00 | |
| S6 | 84,00 | | 82,00 | | 82,00 | | | 78,00 | 82,00 | | 83,00 | |
| S7 | 71,00 | | 71,00 | | 70,00 | | | 68,00 | 71,00 | | 69,00 | |
| S8 | 84,00 | | 79,00 | | 84,00 | | | 89,00 | 99,00 | | 82,00 | |
| S9 | 95,00 | | 95,00 | | 93,00 | | | 96,00 | 99,00 | | 94,00 | |
| mean | 83,56 | | 82,00 | | 83,67 | | | 82,11 | 87,33 | | 83,44 | |
| SD | 8,23 | | 8,26 | | 9,43 | | | 8,75 | 10,31 | | 9,18 | |
| median | 84,00 | | 80,00 | | 82,00 | | | 80,00 | 84,00 | | 82,00 | |
| Variance | 67,78 | | 68,25 | | 89,00 | | | 76,61 | 106,25 | | 84,28 | |

| GLC | | | | | | |
| --- | --- | --- | --- | --- | --- | --- |
| Blood Sample | 1 | 2 | 3 | 4 | 5 | 6 |
| S1 | 5,70 | 6,10 | 5,80 | 5,60 | 5,00 | 5,90 |
| S2 | 5,00 | 5,10 | 4,80 | 4,80 | 4,10 | 5,30 |
| S3 | 4,30 | 5,00 | 4,20 | 4,30 | 3,30 | 4,80 |
| S4 | 4,50 | 5,20 | 4,70 | 4,50 | 3,60 | 4,70 |
| S5 | 6,00 | 5,10 | 5,80 | 5,00 | 4,80 | 5,00 |
| S6 | 4,40 | 4,70 | 4,30 | 4,80 | 5,40 | 4,50 |
| S7 | 5,30 | 5,10 | 5,20 | 4,40 | 4,10 | 4,80 |
| S8 | 5,40 | 4,60 | 5,40 | 5,20 | 4,40 | 4,60 |
| S9 | 5,10 | 4,30 | 5,20 | 4,60 | 4,00 | 4,30 |
| mean | 5,08 | 5,02 | 5,04 | 4,80 | 4,30 | 4,88 |
| SD | 0,59 | 0,50 | 0,59 | 0,42 | 0,67 | 0,48 |
| median | 5,10 | 5,10 | 5,20 | 4,80 | 4,10 | 4,80 |
| Variance | 0,35 | 0,25 | 0,35 | 0,17 | 0,45 | 0,23 |

| WBC | | | | | | | | | | | | | |
| --- | --- | --- | --- | --- | --- | --- | --- | --- | --- | --- | --- | --- | --- |
| Blood Sample | 1 | | 2 | | 3 | | 4 | | 5 | | | 6 | |
| S1 | 9,00 | | 9,20 | | 8,60 | | 9,80 | | 8,90 | | | 8,90 | |
| S2 | 6,10 | | 6,60 | | 5,40 | | 5,90 | | 5,60 | | | 6,10 | |
| S3 | 10,60 | | 11,80 | | 10,50 | | 11,00 | | 9,80 | | | 11,80 | |
| S4 | 6,30 | | 7,10 | | 5,70 | | 6,20 | | 6,60 | | | 5,80 | |
| S5 | 7,50 | | 7,50 | | 6,70 | | 8,50 | | 5,80 | | | 6,80 | |
| S6 | 11,00 | | 13,50 | | 10,80 | | 11,20 | | 10,20 | | | 10,90 | |
| S7 | 5,00 | | 5,90 | | 5,70 | | 5,90 | | 5,20 | | | 6,90 | |
| S8 | 6,10 | | 6,10 | | 6,80 | | 6,80 | | 6,90 | | | 6,40 | |
| S9 | 8,10 | | 9,90 | | 7,50 | | 9,90 | | 7,50 | | | 9,90 | |
| mean | 7,74 | | 8,62 | | 7,52 | | 8,36 | | 7,39 | | | 8,17 | |
| SD | 2,11 | | 2,68 | | 2,03 | | 2,20 | | 1,85 | | | 2,25 | |
| median | 7,50 | | 7,50 | | 6,80 | | 8,50 | | 6,90 | | | 6,90 | |
| Variance | 4,44 | | 7,16 | | 4,14 | | 4,84 | | 3,42 | | | 5,09 | |
| RBC | | | | | | | | | | | | |  |
| Blood Sample | | 1 | | 2 | | 3 | | 4 | | 5 | 6 | |  |
| S1 | | 6,03 | | 6,08 | | 5,51 | | 5,17 | | 5,29 | 5,66 | |  |
| S2 | | 5,74 | | 6,02 | | 5,34 | | 5,55 | | 5,70 | 5,55 | |  |
| S3 | | 5,86 | | 6,09 | | 5,75 | | 5,67 | | 5,88 | 5,88 | |  |
| S4 | | 5,66 | | 6,02 | | 5,57 | | 5,98 | | 5,64 | 5,67 | |  |
| S5 | | 5,12 | | 6,20 | | 5,01 | | 4,81 | | 5,49 | 5,10 | |  |
| S6 | | 7,12 | | 7,17 | | 6,26 | | 5,51 | | 5,65 | 6,36 | |  |
| S7 | | 6,59 | | 6,09 | | 5,38 | | 6,74 | | 5,66 | 7,03 | |  |
| S8 | | 5,31 | | 6,01 | | 4,99 | | 4,97 | | 5,55 | 5,12 | |  |
| S9 | | 5,71 | | 5,01 | | 5,07 | | 5,22 | | 5,07 | 5,22 | |  |
| mean | | 5,90 | | 6,08 | | 5,43 | | 5,51 | | 5,55 | 5,73 | |  |
| SD | | 0,62 | | 0,54 | | 0,41 | | 0,59 | | 0,24 | 0,63 | |  |
| median | | 5,74 | | 6,08 | | 5,38 | | 5,51 | | 5,64 | 5,66 | |  |
| Variance | | 0,38 | | 0,29 | | 0,17 | | 0,34 | | 0,06 | 0,40 | |  |

| Neu | | | | | | |
| --- | --- | --- | --- | --- | --- | --- |
| Blood Sample | 1 | 2 | 3 | 4 | 5 | 6 |
| S1 | 6,20 | 5,50 | 6,40 | 5,70 | 5,80 | 6,50 |
| S2 | 4,10 | 3,80 | 3,40 | 3,30 | 3,70 | 3,40 |
| S3 | 6,00 | 6,80 | 6,70 | 6,30 | 5,90 | 6,70 |
| S4 | 4,70 | 4,50 | 3,80 | 3,50 | 3,80 | 3,80 |
| S5 | 4,30 | 5,40 | 4,50 | 4,70 | 3,70 | 5,00 |
| S6 | 7,90 | 10,00 | 8,40 | 9,00 | 9,00 | 8,20 |
| S7 | 3,60 | 3,00 | 3,00 | 3,40 | 2,90 | 4,00 |
| S8 | 4,00 | 3,50 | 3,40 | 3,30 | 3,40 | 3,50 |
| S9 | 4,10 | 5,60 | 4,20 | 4,30 | 3,90 | 5,60 |
| mean | 4,99 | 5,34 | 4,87 | 4,83 | 4,68 | 5,19 |
| SD | 1,41 | 2,12 | 1,86 | 1,91 | 1,92 | 1,68 |
| median | 4,30 | 5,40 | 4,20 | 4,30 | 3,80 | 5,00 |
| Variance | 2,00 | 4,49 | 3,46 | 3,64 | 3,69 | 2,83 |

| PLT | | | | | | | | | | | | | | | | | | |
| --- | --- | --- | --- | --- | --- | --- | --- | --- | --- | --- | --- | --- | --- | --- | --- | --- | --- | --- |
| Blood Sample | | 1 | | | 2 | | 3 | | | 4 | | | 5 | | | 6 | | |
| S1 | | 208,00 | | | 183,00 | | 213,00 | | | 201,00 | | | 215,00 | | | 176,00 | | |
| S2 | | 130,00 | | | 109,00 | | 131,00 | | | 128,00 | | | 133,00 | | | 121,00 | | |
| S3 | | 220,00 | | | 172,00 | | 213,00 | | | 193,00 | | | 208,00 | | | 214,00 | | |
| S4 | | 160,00 | | | 150,00 | | 177,00 | | | 169,00 | | | 212,00 | | | 194,00 | | |
| S5 | | 219,00 | | | 170,00 | | 228,00 | | | 191,00 | | | 167,00 | | | 231,00 | | |
| S6 | | 244,00 | | | 245,00 | | 254,00 | | | 284,00 | | | 268,00 | | | 217,00 | | |
| S7 | | 230,00 | | | 147,00 | | 255,00 | | | 168,00 | | | 273,00 | | | 230,00 | | |
| S8 | | 190,00 | | | 150,00 | | 171,00 | | | 170,00 | | | 161,00 | | | 131,00 | | |
| S9 | | 170,00 | | | 133,00 | | 161,00 | | | 153,00 | | | 161,00 | | | 123,00 | | |
| mean | | 196,78 | | | 162,11 | | 200,33 | | | 184,11 | | | 199,78 | | | 181,89 | | |
| SD | | 37,19 | | | 38,17 | | 42,89 | | | 43,58 | | | 48,74 | | | 46,01 | | |
| median | | 208,00 | | | 150,00 | | 213,00 | | | 170,00 | | | 208,00 | | | 194,00 | | |
| Variance | | 1383,44 | | | 1457,11 | | 1839,25 | | | 1899,11 | | | 2375,69 | | | 2117,11 | | |
| CPK | | | | | | | | | | | | | | | | | | |
| Blood Sample | 1 | | | 2 | | | | 3 | | | 4 | | | 5 | | | 6 | |
| S1 | 490,00 | | | 348,00 | | | | 516,00 | | | 231,00 | | | 290,00 | | | 190,00 | |
| S2 | 210,00 | | | 163,00 | | | | 185,00 | | | 169,00 | | | 206,00 | | | 163,00 | |
| S3 | 386,00 | | | 329,00 | | | | 374,00 | | | 229,00 | | | 274,00 | | | 179,00 | |
| S4 | 286,00 | | | 201,00 | | | | 276,00 | | | 220,00 | | | 251,00 | | | 136,00 | |
| S5 | 510,00 | | | 373,00 | | | | 502,00 | | | 305,00 | | | 340,00 | | | 149,00 | |
| S6 | 430,00 | | | 368,00 | | | | 418,00 | | | 317,00 | | | 370,00 | | | 368,00 | |
| S7 | 356,00 | | | 252,00 | | | | 344,00 | | | 252,00 | | | 300,00 | | | 175,00 | |
| S8 | 693,00 | | | 535,00 | | | | 706,00 | | | 273,00 | | | 373,00 | | | 387,00 | |
| S9 | 700,00 | | | 513,00 | | | | 642,00 | | | 363,00 | | | 402,00 | | | 390,00 | |
| mean | 451,22 | | | 342,44 | | | | 440,33 | | | 262,11 | | | 311,78 | | | 237,44 | |
| SD | 167,54 | | | 126,67 | | | | 168,51 | | | 58,91 | | | 64,22 | | | 109,49 | |
| median | 430,00 | | | 348,00 | | | | 418,00 | | | 252,00 | | | 300,00 | | | 179,00 | |
| Variance | 28070,44 | | | 16044,03 | | | | 28394,50 | | | 3469,86 | | | 4124,69 | | | 11988,28 | |
| LDH | | | | | | | | | | | | | | | | | | |
| Blood Sample | | | 1 | | | 2 | | | 3 | | | 4 | | | 5 | | | 6 |
| S1 | | | 229,00 | | | 186,00 | | | 227,00 | | | 151,00 | | | 174,00 | | | 152,00 |
| S2 | | | 131,00 | | | 116,00 | | | 132,00 | | | 102,00 | | | 118,00 | | | 108,00 |
| S3 | | | 201,00 | | | 179,00 | | | 193,00 | | | 167,00 | | | 188,00 | | | 157,00 |
| S4 | | | 155,00 | | | 154,00 | | | 166,00 | | | 153,00 | | | 170,00 | | | 130,00 |
| S5 | | | 236,00 | | | 196,00 | | | 239,00 | | | 187,00 | | | 203,00 | | | 163,00 |
| S6 | | | 180,00 | | | 159,00 | | | 176,00 | | | 157,00 | | | 176,00 | | | 140,00 |
| S7 | | | 176,00 | | | 166,00 | | | 181,00 | | | 155,00 | | | 186,00 | | | 142,00 |
| S8 | | | 253,00 | | | 194,00 | | | 261,00 | | | 173,00 | | | 201,00 | | | 167,00 |
| S9 | | | 214,00 | | | 177,00 | | | 210,00 | | | 159,00 | | | 191,00 | | | 149,00 |
| mean | | | 197,22 | | | 169,67 | | | 198,33 | | | 156,00 | | | 178,56 | | | 145,33 |
| SD | | | 40,06 | | | 24,86 | | | 40.02 | | | 23,27 | | | 25,42 | | | 18,18 |
| median | | | 201,00 | | | 177,00 | | | 193,00 | | | 157,00 | | | 186,00 | | | 149,00 |
| Variance | | | 1604,44 | | | 618,25 | | | 1601.5 | | | 541,50 | | | 646,03 | | | 330,50 |

| ASAT | | | | | | |
| --- | --- | --- | --- | --- | --- | --- |
| Blood Sample | 1 | 2 | 3 | 4 | 5 | 6 |
| S1 | 35,00 | 31,00 | 36,00 | 30,00 | 29,00 | 24,00 |
| S2 | 23,00 | 17,00 | 19,00 | 16,00 | 17,00 | 16,00 |
| S3 | 29,00 | 25,00 | 28,00 | 25,00 | 29,00 | 19,00 |
| S4 | 28,00 | 24,00 | 27,00 | 25,00 | 28,00 | 23,00 |
| S5 | 43,00 | 38,00 | 47,00 | 30,00 | 31,00 | 17,00 |
| S6 | 22,00 | 18,00 | 22,00 | 18,00 | 27,00 | 16,00 |
| S7 | 23,00 | 18,00 | 22,00 | 17,00 | 24,00 | 17,00 |
| S8 | 35,00 | 30,00 | 36,00 | 26,00 | 27,00 | 24,00 |
| S9 | 39,00 | 34,00 | 37,00 | 23,00 | 24,00 | 25,00 |
| mean | 30,77 | 26,11 | 30,44 | 23,33 | 26,22 | 20,11 |
| SD | 7,59 | 7,61 | 9,15 | 5,29 | 4,15 | 3,82 |
| median | 29,00 | 25,00 | 28,00 | 25,00 | 27,00 | 19,00 |
| Variance | 57,69 | 57,86 | 83,77 | 28,00 | 17,19 | 14,61 |

| PAL | | | | | | |
| --- | --- | --- | --- | --- | --- | --- |
| Blood Sample | 1 | 2 | 3 | 4 | 5 | 6 |
| S1 | 97,00 | 101,00 | 105,00 | 105,00 | 99,00 | 105,00 |
| S2 | 79,00 | 78,00 | 79,00 | 83,00 | 84,00 | 82,00 |
| S3 | 73,00 | 74,00 | 79,00 | 81,00 | 81,00 | 82,00 |
| S4 | 61,00 | 64,00 | 67,00 | 71,00 | 72,00 | 67,00 |
| S5 | 55,00 | 56,00 | 59,00 | 50,00 | 51,00 | 50,00 |
| S6 | 70,00 | 72,00 | 77,00 | 71,00 | 72,00 | 70,00 |
| S7 | 68,00 | 73,00 | 77,00 | 59,00 | 61,00 | 69,00 |
| S8 | 57,00 | 57,00 | 59,00 | 58,00 | 61,00 | 57,00 |
| S9 | 76,00 | 77,00 | 79,00 | 77,00 | 73,00 | 77,00 |
| mean | 70,67 | 72,44 | 75,67 | 72,78 | 72,67 | 73,22 |
| SD | 12,89 | 13,46 | 13.82 | 16,44 | 14,29 | 16,00 |
| median | 70,00 | 73,00 | 77,00 | 71,00 | 72,00 | 70,00 |
| Variance | 166,25 | 181,28 | 191,00 | 270,19 | 204,25 | 255,94 |

| CRP | | | | | | |
| --- | --- | --- | --- | --- | --- | --- |
| Blood Sample | 1 | 2 | 3 | 4 | 5 | 6 |
| S1 | 2,40 | 2,40 | 2,50 | 2,20 | 2,00 | 1,70 |
| S2 | 0,40 | 0,30 | 0,30 | 0,30 | 0,30 | 0,40 |
| S3 | 1,90 | 1,80 | 1,90 | 1,80 | 1,90 | 1,80 |
| S4 | 0,20 | 0,20 | 0,30 | 0,40 | 0,30 | 0,40 |
| S5 | 0,90 | 0,80 | 0,90 | 0,70 | 0,80 | 0,40 |
| S6 | 2,80 | 2,80 | 2,90 | 2,60 | 2,70 | 2,20 |
| S7 | 0,90 | 0,80 | 1,10 | 0,40 | 0,40 | 1,00 |
| S8 | 1,90 | 1,80 | 1,80 | 1,90 | 1,90 | 1,70 |
| S9 | 1,70 | 1,60 | 1,60 | 1,50 | 1,60 | 1,60 |
| mean | 1,46 | 1,39 | 1,48 | 1,31 | 1,32 | 1,24 |
| SD | 0,90 | 0,91 | 0,91 | 0,88 | 0,89 | 0,70 |
| median | 1,70 | 1,60 | 1,60 | 1,50 | 1,60 | 1,60 |
| Variance | 0,81 | 0,84 | 0,83 | 0,77 | 0,79 | 0,50 |
