# Supplementary material for: Pomegranate Supplementation Accelerates Recovery of Muscle Damage and Soreness and Inflammatory Markers after a Weightlifting Training Session
Source: PLoS One. 2016 Oct 20;11(10):e0160305. doi: 10.1371/journal.pone.0160305 (PMC5072630; doi:10.1371/journal.pone.0160305)
Supplement: S1 Protocol — (DOCX) [file pone.0160305.s002.docx]

*I confirm that this clinical trial protocol is the version that was submitted to and approved by our ethics committee before the trial began*

**Methods**

The authors confirm that all ongoing and related trials for this intervention are registered.

***Participants***

Nine male elite weightlifters (age: 21 ± 0.5 years, body mass: 80 ± 20 kg, height 175 ± 8.1 cm (mean ± SD)) volunteered to participate in this study. The participants were recruited on the basis of: (i) they trained at least five sessions per week (between 15h:30 and 17h:30) with 90 to 120 min per session,(ii) they had an experience of more than 3 years in Olympic weightlifting and (iii) they didn’t have any injuries and they didn’t use any antioxidant (e.g., vitamin E, A, C etc..) or anti-inflammatory during the experimentation period and one month before. After receiving a thorough explanation of the possible risks and discomforts associated with the experimental procedures, the participants provided written informed consent to take part to the experiment. The study was conducted according to the Declaration of Helsinki. The protocol and the consent form were fully approved by the institutional review board “Habib Bourguiba University hospital ethics committee before the commencement of the assessments.

***Experimental design***

One week before the start of the experimental period (01/01/2015), the heaviest weight lifted in a single repetition (1-RM) was assessed for each participant in each movement (Figure 1). After an ascending warm-up from 40 to 80% of athlete's estimated maximum [22], 1-RM was determinate in three trials with large recovery time (5 min) as following: 1^st^ sets (8–10 reps) × (load 50% 1-RM e) – 5’ of recovery – 2^nd^ sets (3–5 reps) × (load 75% 1-RM e) 5’ recovery – 3^rd^ sets (1–3 reps) × (load 90% 1-RM e). As suggested by Kraemer and Fry. [23], they emphasise that on-going encouragement and communication with the athletes during this testing is crucial to obtain the best performance. Also, estimated 1-RM will have to be verified in the next two days. Then participants performed-as part of their habitual training program from 08h:00 to 09h:45- two training sessions (Figure 1) using respectively, PLA and POMj supplementations (500ml), with a recovery period of 48 h in between (07/01/2015 and 09/01/2015). Upon arrival for their first test session, each participant's body mass (Tanita, Tokyo, Japan) and height were recorded. Moreover, before and after each tested training session, oral temperature was recorded with a calibrated digital clinical thermometer (Omron, Paris, France; accuracy: 0.05°C) inserted sublingually for at least 3 min with the subjects in a seated resting position for at least 15 min. Also, before and after each training session, fasting blood samples (blood sample 2, 3, 4 and 5, Figure1) were collected and heart rate (HR) and systolic arterial pressure (SAP) were recorded using a heart rate monitor and a manual sphygmomanometer. Additionally, to assess the recovery kinetic of the biological parameters, blood sample, temperature, HR and SAP were collected at resting state (i.e., after 10 days of recovery, blood sample 6) and immediately (3min) after the training session which proceed the PLA session (blood sample 1). The RPE was recorded after each training session [24]. Before test session, participants were fasting and allowed to drink only one glass of water (15–20 cl) to avoid the effects of postprandial thermogenesis [25]. It should be noted that based on the results of Petterson et al. 2008 who showed that values of markers of muscle damage remain raised for at least 7days following intensive weightlifting exercises, a recovery period of 10 days was chosen to evaluate the biological resting state (19/01/2015). Additionally, given that (i) using randomized order in this study will results in consuming POMj then PLA supplementations (after 48h) for some participant and (ii) the delayed effect of POMj (which we expect) could alter the results of PLA supplementation, authors in the present study choose to avoid randomized order and to evaluate the PLA at first then the POMj effect using all the participant together.


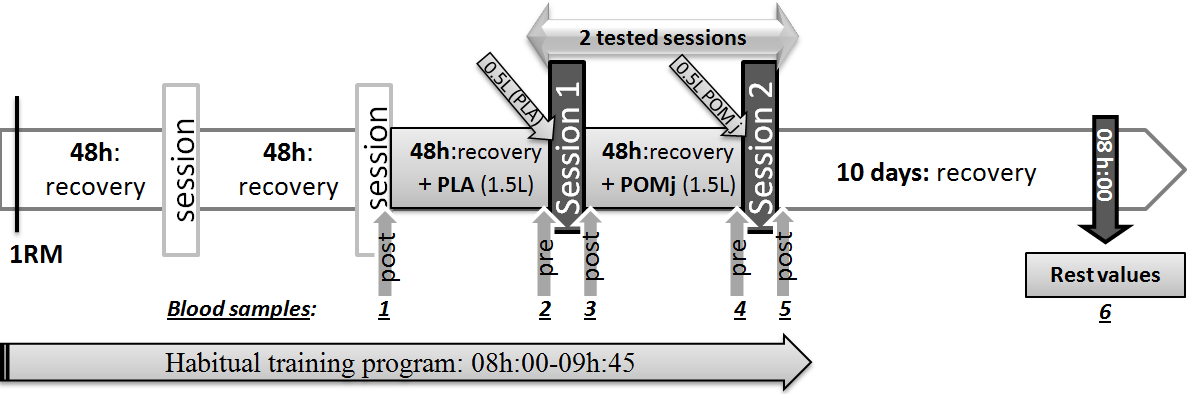


**Figure I:** Experimental design

***Training session***

Each training session includes three Olympic-Weightlifting exercises: snatch, clean and jerk, and the squat [6, 7, 26]. After increasing the load until unsuccessful trial in the power snatch and power clean and jerk, five sets for each exercises were performed (i.e., two sets at 85% of 1-RM with three repetitions per set and three sets at 90% of 1- RM with two reps per set) with a passive recovery period of 5 min in-between [27]. Each session was preceded by 10 min warm-up wherein the participants will perform 3–5 repetitions with increasing loads (i.e., from 40 to 80% of the 1-RM). The performance in each session was measured by the total volume lifted in the two Olympic movements (volume lifted (kg) in snatch added volume lifted (kg) in clean and Jerk: only the right lifts are taken into consideration) [6, 7] and by the maximal power lifted amounts in both Olympic movement (i.e., the maximal load lifted (kg) in the power snatch added to the maximal load lifted (kg) in the power clean and Jerk).

***Rating of Perceived Exertion (RPE) Scale***

The RPE scale (15-points) runs from 6 (very, very light) to 20 (very, very hard) 10. RPE scale measures feelings of effort, strain, discomfort, and/or fatigue experienced during physical task. Although this is a subjective measure (person's exertion), RPE values were shown to provide good estimation of the increase in heart rate, muscle fatigue and several other physiological measures during physical activity [24].

***Delayed onset muscle soreness (DOMS)***

Delayed onset soreness (DOMS) of the knee extensor and elbow flexor was determined 48 hours after training sessions. 48h after each training session, participants were asked to rate subjectively the degree of soreness in both muscles using a visual analog scale of 0 (absence of soreness) to 10 (unbearable soreness) [28]. Soreness was normalized to 100% of the maximal perceived level.

***Dietary records***

To assess the adequacy of nutrient intake, a consecutive dietary record over 7 days was completed. All participants received a detailed verbal explanation and written instructions on data collection procedures. Participants were asked to continue with their usual dietary habits during the period of dietary recording and to be as accurate as possible in recording the amounts and types of food and fluid consumed. A list of common household measures, such as cups and tablespoons, and specific information about the quantity in each measurement (grams, etc.) were given to each participant. Each individual's diet was calculated using the Bilnut 4 software package (SCDA Nutrisoft, Cerelles, France) and the food composition tables published by the Tunisian National Institute of Statistics in 1978.

***Pomegranate and placebo supplementations***

Supplements (1500ml) of placebo (PLA) or pomegrenate juice (POMj) were taken three times daily in the 48h which proceed the first and the second tested training sessions respectively (i.e. 250ml × 6 times with 8-h intervals between it). Moreover, during these tested sessions, subject consumed an additional 500ml of PLA and 500ml of POMj, respectively (Figure 1). The tested quantity of the natural POMj were prepared from a fresh pomegranate fruit 48h before the beginning of the experimentation and were shipped frozen and stored at -4°C. No additional chemical products were added to the natural POMj. Each 500-mL of the tested POMj contained 2.56g of total polyphenol, 1.08g of orthodiphenols, 292.59mg of flavonoids and 46.75mg of flavonols. Subjects were reminded verbally through phone communication to consume at the required times their supplements. Placebo juice consisted of an Pomegranate-flavored commercial drink contained water, citric acid, natural flavor and natural identical flavor (Pomegranate), sweeteners (aspartame × (0.3g/l), acesulfame K (0.16g/l)), stabilizers (Arabic gum) and lacked antioxidants, fruit and vegetable extracts or vitamins. Placebo juice contains no polyphenols.

Given that the daily drink of 500ml (2 × 250ml/day) pomegranate juice for five and either fifteen days prior to an intense exercise show contradictory effect in the recovery levels of muscle damage, muscle strength and muscle soreness [3,20] and given that the present study investigate the effect of the natural POMj consumed only 2 days before the training session. A supplementation quantity of 750ml (3 × 250ml /day) has been chosen.

***Phenolic compounds***

*Extraction of phenolic fraction*

The phenolic extracts were obtained following the procedure of Chtourou et al. [29] with some modifications. In fact, the oil sample (4g) was added to 2 mL of *n*-hexane and 4 mL of a methanol/water (60:40, v/v) mixture in a 20 mL centrifuge tube. After vigorous mixing, they were centrifuged for 3 min. The hydroalcoholic phase was collected, and the hexane phase was re-extracted twice with 4 mL of the methanol/water (60:40, v/v) solution each time. Finally, the hydroalcoholic fractions were combined, washed with 4 mL of *n*-hexane to remove the residual oil, then concentrated and dried by evaporative centrifuge in vacuum at 35°C.

*Determination of the total phenols and o-diphenols contents*

The determination of the total phenolic compounds was performed by means of the Folin-Ciocalteau reagent using the method described by Gargouri et al. [30]. The total phenolic content was expressed as milligrams of gallic acid (GA) equivalent per kilogram of oil (y = 0.011x, R²= 0.990). The optical density (OD) was measured at λ= 765 nm, using a spectrophotometer (Shimadzu UV-1800 PC, Japan). The concentration of *o*-diphenolic compounds in the methanolic extract was determined by the method of Dridi-Gargouri et al. [31]. The total *o*-diphenolic content was expressed as milligrams of gallic acid (GA) equivalent per kilogram of oil (y = 1.144x, R²= 0.999). The optical density (OD) was measured at λ= 370 nm, using a spectrophotometer (Shimadzu UV-1800 PC, Japan).

*Determination of total flavonoids*

Total flavonoids were measured by a colorimetric assay developed by Gargouri et al. [30]; 1 mL aliquot of appropriately diluted sample or standard solutions of catechin (20, 40, 60, 80 and 100 mg L-1) was added to a 10-mL volumetric flask containing 4 mL double-distillate H_2_O. At zero time, 0.30 mL 5 % NaNO2 was added to the flask. After 5 min, 0.30 mL 10 % AlCl3 was added. At 6 min, 2 mL (1 mol L-1) NaOH was added to the mixture. Immediately, the reaction flask was diluted to volume with the addition of 2.40 mL of double-distillate H_2_O and thoroughly mixed. Concerning the absorbance of the mixture, pink in colour, it was determined at 510 nm versus prepared water blank. As for the total flavonoids of fruits, they were expressed on a fresh weight basis as mg 100 g-1 catechin equivalents (CE). It is worth noting that the samples were analysed in triplicate.

***Blood sampling and analysis***

Blood samples (6ml) were collected six time for each participant (Figure1) from a forearm vein (i.e., 2.5ml in tube contains EDTA for hematological parameters and 3.5ml in tube contains Heparine for GLY, CRE, CRP and muscle damage parameters). At rest (blood sample 6) and before the two tested sessions (blood sample 2 and 4) samples were collected after 5 min of being seated. Immediately after training sessions (blood sample 1, 3 and 5) sample were also collected at 3min of being seated. Samples were placed in an ice bath and centrifuged immediately at 2500 rpm (× g) for 10 min. Aliquots of the resulting plasma were stored at -80°C until analyses.

To eliminate inter-assay variance, all samples were analysed in the same assay run. All assays were performed in duplicate in the same laboratory with simultaneous use of a control serum from Randox. Haematological parameters (i.e., white blood cells (WBC), neutrophils (NEU), red blood cells (RBC), hemoglobin (HGB), hematocrit (HCT)and platelets (PLT) were generally performed within 3 h in a multichannel automated blood cell analyser Beckman Coulter Gen system-2 (Coulter T540). Glycemia (GLY), Creatinine (CRE), muscle damage markers and c-reactive protein (CRP) were determined spectrophotometrically using Architect Ci 4100 d'ABOTT. N-acetyl-L-cysteine method, the hydrolyse of phosphate parametrophenyl method, enzymatique method (using L-gammaglutamyl 3 carboxy 4 nitranilide as a substrate), the oxidation of lactate on pyruvate method and immunoturbidimetric method were respectively used to determine the activities of Creatinine kinase (CK), Alkaline phosphate (PAL), Gammaglutamyl (GGT), Lactate dehydrogenase (LDH) and CRP. The intra-assay coefficient of variation for these parameters kit were respectively 1.3%, 4.6%, 2.3%, 0.2% and 1.16%. Aspartate aminotransferase (ASAT) and alanine aminotransferase (ALAT) activities were determined by measuring NADH oxidation with 1.1 and 1.5% intra-assay coefficient of variation for their kit.

***Statistical analyses***

All statistical tests were processed using STATISTICA Software (Stat-Soft, France). Following normality confirmation using the Shapiro–Wilks W-test. To analyse the effect of POMj supplementation in the performance and RPE paired simple t-test was used. To analyse the effect of POMj supplementation in the biological responses during training sessions (pre-post values) a two-way ANOVA (2 levels [supplementation (PLA and POMj] × 2 levels [training (Pre and Post]) with repeated measures was used. To analyse the effect of POMj supplementation in the recovery kinetic of studied parameters one-way ANOVA was used. Post hoc tests were conducted when significant main effects were found using Fisher's least significant difference (LSD). Effect sizes were calculated as partial eta-squared (η_p_^2^) for the ANOVA analysis and as Cohen's d for the paired sample t-test to assess the practical significance of our findings. Pearson correlation was used to assess the correlation between DOMS and CK values. Significance was set at p < 0.05.
