# Supplementary material for: Pomegranate Supplementation Accelerates Recovery of Muscle Damage and Soreness and Inflammatory Markers after a Weightlifting Training Session
Source: PLoS One. 2016 Oct 20;11(10):e0160305. doi: 10.1371/journal.pone.0160305 (PMC5072630; doi:10.1371/journal.pone.0160305)
Supplement: S1 Receipt of the Trial Registry — (PDF) [file pone.0160305.s003.pdf]

ClinicalTrials.gov Protocol Registration and Results System (PRS) Receipt
Release Date: 03/02/2016

ClinicalTrials.gov ID: NCT02697903

Study Identification

Unique Protocol ID: 16-2015

Brief Title: Pomegranate Improve Biological Recovery Kinetics in Elite Weightlifter

Official Title: Pomegranate Supplementation Accelerates the Recovery Kinetics of Muscle Damage, Muscle Soreness and Inflammatory Marker After Weightlifting Training Session.

Secondary IDs:

Study Status

Record Verification: March 2016

Overall Status: Completed

Study Start: January 2015

Primary Completion: February 2015 [Actual]

Study Completion: February 2015 [Actual]

Sponsor/Collaborators

Sponsor: The Higher Institute of Sport and Physical Education of Sfax

Responsible Party: Principal Investigator
Investigator: Ammar Achraf [aachraf]
Official Title: Dr
Affiliation: The Higher Institute of Sport and Physical Education of Sfax

Collaborators:

Oversight

FDA Regulated?: No

IND/IDE Protocol?: No

Review Board: Approval Status: Approved
Approval Number: 16/2015
Board Name: La comité locale du laboratoire biochimie de l'hôpital habib bourguiba
Board Affiliation: Laboratoire de biochimie, hopital Habib Bourguiba, Sfax, Tunisia
Phone: 0021622135609
Email: mouna.turki@gmail.com

Data Monitoring?: Yes

Plan to Share Data?: Yes
Data will be shared with researchers outside the primary research group for reanalysis purpose.

Oversight Authorities: Tunisia: La comité locale du laboratoire biochimie de l'hôpital habib bourguiba

Study Description

Brief Summary: From the recent scientific literature in the field of biological response to physical exercise, a significant increase in muscle damage, soreness and inflammation were registered immediately and even 48 after resistance exercise. In the other hand, from the recent scientific literature in the field of nutrition it is well established that Pomegranate juice is a potent antioxidant that can help prevent or treat various disease risk factors. However, only few studies associated the effect of the pomegranate with the physical exercise. To the authors' knowledge, there are no studies investigated the acute and delayed effect of pomegranate supplementation in performance and muscle recovery after exercises involved muscle of whole body. Therefore, the aim of the present study was to investigate the effect of natural Pomegranate juice supplementation on the acute and delayed response of muscle soreness and biochemical parameters following weightlifting training session

Detailed Description: Participants (i.e., 9 elite weightlifters) performed-as part of their habitual training program from 08h:00 to 09h:45- two training sessions using respectively, PLA and POMj supplementations (500ml), with a recovery period of 48 h in between. Upon arrival for their first test session, each participant's body mass and height were recorded. Moreover, before and after each tested training session, oral temperature was recorded with a calibrated digital clinical thermometer (Omron, Paris, France; accuracy: 0.05°C) inserted sublingually for at least 3 min with the subjects in a seated resting position for at least 15 min. Also, before and after each training session, fasting blood samples (blood sample 2, 3, 4 and 5, Figure1) were collected and heart rate (HR) and systolic arterial pressure (SAP) were recorded using a heart rate monitor and a manual sphygmomanometer. Additionally, to assess the recovery kinetic of the biological parameters, blood sample, temperature, HR and SAP were collected at resting state (i.e., after 10 days of recovery, blood sample 6) and immediately (3min) after the training session which proceed the PLA session (blood sample 1). Given that values of markers of muscle damage remain raised for at least 7 days following intensive weightlifting exercises, a recovery period of 10 days was chosen to evaluate the biological resting state. Additionally, given that (i) using randomized order in this study will results in consuming POMj then PLA supplementations (after 48h) for some participant and (ii) the delayed effect of POMj (which we expect) could alter the results of PLA supplementation, in the present study investigators choose to avoid randomized order and to evaluate the PLA at first then the POMj effect using all the participant together. Supplements (1500ml) of placebo (PLA) or pomegrenate juice (POMj) were taken three times daily in the 48h which proceed the first and the second tested training sessions respectively (i.e. 250ml × 6 times with 8-h intervals between it). Moreover, during these tested sessions, subject consumed an additional 500ml of PLA and 500ml of POMj, respectively. The tested quantity of the natural POMj were prepared from a fresh pomegranate fruit 48h before the beginning of the experimentation and were shipped frozen and stored at -4°C. No additional chemical products were added to the natural POMj. Each 500-mL of the tested POMj contained 2.56g of total polyphenol, 1.08g of ortho-diphenols, 292.59mg of flavonoids and 46.75mg of flavonols. Subjects were reminded verbally through phone communication to consume at the required times their supplements. Placebo juice consisted of an Pomegranate-flavored commercial drink contained water, citric acid, natural flavor and natural identical flavor (Pomegranate), sweeteners (aspartame × (0.3g/l), acesulfame K (0.16g/l)), stabilizers (Arabic gum) and lacked antioxidants, fruit and vegetable extracts or vitamins. Placebo juice contains no polyphenols.

Conditions

Conditions: Healthy

Keywords: Creatine kinase supplementation
C-reactive protein
resistance training
Pomegranate supplementation
pomegranate juice
muscle damage response

Study Design

Study Type:	Interventional
Primary Purpose:	Supportive Care
Study Phase:	N/A
Intervention Model:	Crossover Assignment
Number of Arms:	2
Masking:	Open Label
Allocation:	Non-Randomized
Endpoint Classification:	Efficacy Study
Enrollment:	9 [Actual]

Arms and Interventions

Arms	Assigned Interventions
Experimental: 9 elite weightlifters<br>age: 21 ± 0.5 years, body mass: 80 ± 20 kg, height 175 ± 8.1 cm (mean ± SD). They received "Natural Pomegranate Juice" supplementation during and 48h following the first weightlifting training session.	Dietary Supplement: Natural Pomegranate Juice<br>Supplements (1500ml) of pomegrenate juice (POMj) were taken three times daily in the 48h which proceed the first tested training sessions (i.e. 250ml × 6 times with 8-h intervals between it). Moreover, during this tested sessions, subject consumed an additional 500ml of POMj. Arm-specific: Experimental: 9 elite weightlifter
Placebo Comparator: 9elite weightlifters<br>age: 21 ± 0.5 years, body mass: 80 ± 20 kg, height 175 ± 8.1 cm (mean ± SD). They received "Placebo Juice" supplementation during and 48h following the second weightlifting training session.	Dietary Supplement: Placebo Juice<br>Supplements (1500ml) of Placebo juice (PLA) were taken three times daily in the 48h which proceed the second tested training sessions (i.e. 250ml × 6 times with 8-h intervals between it). Moreover, during this tested sessions, subject consumed an additional 500ml of POMj. Arm-specific: Placebo Comparator: 9 elite weightlifter

Outcome Measures

Primary Outcome Measure:

1. Change in physical performance from using Placebo to using Pomegranate juice
   [Time Frame: 3min after Placebo and Pomegranate training sessions] [Safety Issue: No]
   The performance in each session was measured by the total volume lifted in the two Olympic movements (volume lifted (kg) in snatch added volume lifted (kg) in clean and Jerk: only the right lifts are taken into consideration) and by the maximal power lifted amounts in both Olympic movement (i.e., the maximal load lifted (kg) in the power snatch added to the maximal load lifted (kg) in the power clean and Jerk)

2. Change in Acute Hematological responses from using Placebo to using Pomegranate juice
   [Time Frame: 3min after Placebo and Pomegranate training sessions] [Safety Issue: No]

Haematological parameters (i.e., white blood cells (WBC), neutrophils (NEU), red blood cells (RBC), hemoglobin (HGB), hematocrit (HCT)and platelets (PLT) were generally performed within 3 h of blood sampling in a multichannel automated blood cell analyser Beckman Coulter Gen system-2 (Coulter T540)

3. Change in Acute Biochemical responses from using Placebo to using Pomegranate juice
   [Time Frame: 3min after Placebo and Pomegranate training sessions] [Safety Issue: No]
   Glycemia (GLY), Creatinine (CRE), muscle damage markers (Creatinine kinase (CK), Alkaline phosphate (PAL), Gammaglutamyl (GGT), Lactate dehydrogenase (LDH)) and c-reactive protein (CRP) were determined spectrophotometrically using Abott Architect Ci 4100

4. Change in Delayed Hematological responses from using Placebo to using Pomegranate juice
   [Time Frame: 48h after Placebo and Pomegranate training sessions] [Safety Issue: No]
   Haematological parameters (i.e., white blood cells (WBC), neutrophils (NEU), red blood cells (RBC), hemoglobin (HGB), hematocrit (HCT)and platelets (PLT) were generally performed within 3 h of blood sampling in a multichannel automated blood cell analyser Beckman Coulter Gen system-2 (Coulter T540)

5. Change in Delayed Biochemical responses from using Placebo to using Pomegranate juice
   [Time Frame: 48h after Placebo and Pomegranate training sessions] [Safety Issue: No]
   Glycemia (GLY), Creatinine (CRE), muscle damage markers (Creatinine kinase (CK), Alkaline phosphate (PAL), Gammaglutamyl (GGT), Lactate dehydrogenase (LDH)) and c-reactive protein (CRP) were determined spectrophotometrically using Abott Architect Ci 4100

Secondary Outcome Measure:

6. Change in the acute fatigue perception (RPE values) from Placebo to Pomegranate supplementation
   [Time Frame: 3min after Placebo and Pomegranate training sessions] [Safety Issue: No]
   The RPE scale (15-points) runs from 6 (very, very light) to 20 (very, very hard) 10. RPE scale measures feelings of effort, strain, discomfort, and/or fatigue experienced during physical task. Although this is a subjective measure (person's exertion), RPE values were shown to provide good estimation of the increase in heart rate, muscle fatigue and several other physiological measures during physical activity

7. Change in the delayed muscle soreness (DOMS values) from Placebo to Pomegranate supplementation
   [Time Frame: 48 after Placebo and Pomegranate training sessions] [Safety Issue: No]
   Delayed onset soreness (DOMS) of the knee extensor and elbow flexor was determined 48 hours after training sessions. 48h after each training session, participants were asked to rate subjectively the degree of soreness in both muscles using a visual analog scale of 0 (absence of soreness) to 10 (unbearable soreness) [28]. Soreness was normalized to 100% of the maximal perceived level.

8. Change in the acute oral temperature response from Placebo to Pomegranate supplementation
   [Time Frame: 3min after Placebo and Pomegranate training sessions] [Safety Issue: No]
   oral temperature was recorded with a calibrated digital clinical thermometer (Omron, Paris, France; accuracy: 0.05°C) inserted sublingually for at least 3 min with the subjects in a seated resting position

9. Change in the acute Heart rate (HR) response from Placebo to Pomegranate supplementation
   [Time Frame: 3min after Placebo and Pomegranate training sessions] [Safety Issue: No]
   heart rate (HR) was recorded using a heart rate monitor

10. Change in the acute systolic arterial pressure (SAP) response from Placebo to Pomegranate supplementation
    [Time Frame: 3min after Placebo and Pomegranate training sessions] [Safety Issue: No]
    systolic arterial pressure (SAP) was recorded using a manual sphygmomanometer

11. Baseline values of body mass
    [Time Frame: at baseline status] [Safety Issue: No]

12. baseline values of height
    [Time Frame: at baseline status] [Safety Issue: No]

Eligibility

Minimum Age: 20 Years

Maximum Age: 24 Years

Gender: Male

Accepts Healthy Volunteers?: Yes

Criteria: Inclusion Criteria:

- trained at least five sessions per week with 90 to 120 min per session
- had an experience of more than 3 years in Olympic weightlifting

Exclusion Criteria:

- use any antioxidant (e.g., vitamin E, A, C etc..) or anti-inflammatory during the experimentation period and one month before
- had injuries during the experimentation period and one month before

Contacts/Locations

Study Officials: Achraf Ammar, Dr
Study Principal Investigator
Institute of sport and physical education of Sfax, Tunisia

Locations: Tunisia
Achraf
Sfax, Tunisia, 3000

References

Citations:

Links:

Study Data/Documents:

U.S. National Library of Medicine | U.S. National Institutes of Health | U.S. Department of Health & Human Services
